# It's not just what you have, but when: The role and timing of developmental assets in the mental health of young adults

**DOI:** 10.1111/jora.70217

**Published:** 2026-07-01

**Authors:** Geneviève Morneau‐Vaillancourt, Mélodie Thibault, Alexe Bilodeau‐Houle, Maude Comtois‐Cabana, Michel Boivin, Sylvana Côté, Marie‐Claude Geoffroy, Alexandra Matte‐Landry, Isabelle Ouellet‐Morin

**Affiliations:** ^1^ École de criminologie Université de Montréal Montréal Québec Canada; ^2^ Centre de recherche de l'Institut universitaire en santé mentale de montréal Université de Montréal Montréal Québec Canada; ^3^ Faculty of Health Sciences Simon Fraser University Burnaby British Columbia Canada; ^4^ Centre for Molecular Medicine and Therapeutics BC Children's Hospital Research Institute Vancouver British Columbia Canada; ^5^ Département de psychologie Université de Montréal Montréal Québec Canada; ^6^ École de psychologie Université Laval Québec City Québec Canada; ^7^ École de santé publique Université de Montréal Montréal Québec Canada; ^8^ Centre de recherche du Centre hospitalier universitaire Sainte‐Justine Montréal Québec Canada; ^9^ Department of Psychiatry McGill University Montréal Québec Canada; ^10^ Douglas Research Center Montréal Québec Canada; ^11^ École de travail social et de criminologie Université Laval Québec City Québec Canada

**Keywords:** anxiety, depression, developmental assets, developmental timing, happiness, life satisfaction, life‐course theory

## Abstract

As the mental health of young adults is declining, finding effective strategies to support them is crucial. Young people who possess, or are exposed to, more developmental assets report higher well‐being and fewer mental problems. Developmental assets are promotive factors that include both individual strengths, like strong social skills and positive identity, and external resources, like supportive relationships and safe living conditions. However, no longitudinal study has yet examined when in development the presence of developmental assets is most impactful. This study aimed to identify which life‐course hypotheses—sensitive period, accumulation, or recency—best explain the associations between developmental assets across childhood and adolescence and mental health in emerging adulthood. Data were drawn from 1344 White, French‐speaking participants of the Quebec Longitudinal Study of Child Development (57% female), a population‐based cohort of children born in the Province of Quebec in 1997/1998 and followed up until now. Developmental assets, measured as both broad dimensions (internal and external) and specific categories (e.g., social competencies, support), were assessed across development from ages 5 (2003) to 17 years (2015) using reports from mothers, teachers, interviewers, and the youth themselves. Self‐reports of life satisfaction, happiness, and symptoms of anxiety and depression were collected twice, at ages 19 (2017) and 22 years (2020). Although all effect sizes were small, findings from the Structured Life‐Course Modeling Approach showed that both internal and external assets present later in adolescence were most beneficial in supporting life satisfaction, thereby supporting the recency hypothesis. In contrast, a sensitive period hypothesis best explained the associations between external assets and both anxiety and depression symptoms, with assets present during the transition to secondary school (age 12 years) showing the strongest associations. When examining specific asset categories, social competencies, positive identity, support from families, schools, and neighborhoods, and safety emerged as especially salient for later mental health. This study highlights the importance of understanding when and which assets matter most to inform more developmentally targeted approaches promoting youth well‐being.

## INTRODUCTION

Emerging adulthood is a developmental period characterized by increased independence and autonomy, yet it is also a time of heightened vulnerability to mental health problems. Approximately 75% of individuals receive their first diagnosis of a mental disorder before the age of 25 (Arnett, 2007; Solmi et al., 2022). Over the past two decades, young adults have experienced increasing levels of anxiety and depression, especially young women, while reporting declining happiness and life satisfaction (Cybulski et al., 2021; Dykxhoorn et al., 2024; Marquez et al., 2024; McGorry et al., 2024; Twenge et al., 2019; Twenge & Blanchflower, 2025). According to the dual mental health framework, mental health is not merely the absence of illness, but also the presence of well‐being, such as positive emotions, happiness, and life satisfaction (Keyes, 2005; Magalhães, 2024; Suldo & Shaffer, 2008; World Health Organization, 2022). In light of these trends, the decline in young adults' mental health underscores the need to identify everyday promotive factors that both (1) mitigate mental distress and (2) enhance mental well‐being.

According to resilience theory, promotive factors support mental health regardless of whether risk is present, in comparison with protective factors, which specifically buffer or moderate the effects of risk factors in contexts of vulnerability (Fergus & Zimmerman, 2005; Yule et al., 2019). Identifying promotive factors that emphasize youth strengths (i.e., strength‐based approach), as opposed to focusing on deficits or vulnerabilities, can benefit all youths, as they can be incorporated into school, family, and community programs without the need to screen for risk first (Saleebay, 1996). Meta‐analyses and systematic reviews have identified a wide range of evidence‐based promotive factors that support mental health, including lifestyle habits (e.g., consistent physical activity, healthy sleep and diet and contact with nature), supportive social relationships, community resources, as well as individual factors, such as academic achievement, school engagement, adaptive coping skills, personality traits, and psychological assets such as hope and gratitude (Bratman et al., 2021; Cairns et al., 2014; Magalhães, 2024; Zimmermann et al., 2020). These findings suggest that strong mental health arises from the cumulative effects of multiple promotive factors working together, rather than from the influence of any single factor in isolation.

This perspective aligns with ecological models of development, which emphasize the cumulative and nested influences of societal, cultural, individual (e.g., genetics), and environmental risk factors (Bronfenbrenner, 1994; Sameroff, 2010). One ecological model particularly well suited to understanding how promotive factors support youth mental health is the Developmental Assets framework (Benson et al., 2011; Scales et al., 2000; Syvertsen et al., 2021). Grounded in research on prevention, resilience, and positive youth development, the framework identifies 40 assets that foster young people's thriving and flourishing. Assets are organized into distinct categories, grouped under two overarching dimensions: internal and external assets. Individual assets include, for example, commitment to learning and social competencies, whereas external assets include, for example, social support and boundaries and expectations from adults within family, school, peer, and community settings (Benson et al., 2011; Scales et al., 2000; Syvertsen et al., 2021). Although developmental assets do not capture this broad range of influences—and therefore can explain only part of youth mental health—they offer a useful ecological framework for considering simultaneous multiple factors across levels of analyses, from individual characteristics to community‐based contexts that may shape mental health in adulthood. Meanwhile, some limitations of the Developmental Assets framework have been noted. Measurement challenges have been documented for the widely used Attitudes and Behaviors: Profiles of Student Life (A&B) survey, including weaker psychometric properties for some assets, reliance on single‐item indicators, and the lack of reported test–retest reliability estimates over short intervals (Benson et al., 2011). Moreover, much of the supporting evidence is cross‐sectional and correlational, limiting support for causal inference. Finally, the empirical evidence remains largely concentrated in U.S. samples (Martin‐Barrado & Gomez‐Baya, 2024), raising questions about generalizability.

Two key findings emerge from research on developmental assets. First, the greater the number of assets to which young people are exposed or possess, the better they tend to function across academic, emotional, social, and behavioral domains (Benson et al., 2011; Martin‐Barrado & Gomez‐Baya, 2024; Soares et al., 2024). Second, developmental assets appear to serve a dual role, both promoting positive development and providing protection against problems (Catalano et al., 2004; Martin‐Barrado & Gomez‐Baya, 2024).

Despite these advances, two significant gaps remain. First, the lack of longitudinal studies limits our understanding of how developmental assets in childhood and adolescence relate to mental health in emerging adulthood. Second, it remains unclear whether the timing of these assets matters, for example, whether their associations with adult mental health vary across developmental periods or are particularly strong at specific time points, such as the transition from primary to secondary school.

Only a handful of longitudinal studies have explored the prospective relationships between developmental assets and various aspects of young people's mental health, such as self‐control and internet gaming disorders in Chinese adolescents (Xiang et al., 2022, 2024; Xiang & Gan, 2022), happiness and depression symptoms in Taiwanese adolescents (Tsai et al., 2021), substance use in adolescents from the Add Health study, a representative sample from the United States (Bleck & DeBate, 2016), and emotional resilience in adolescents from the Project on Human Development in Chicago Neighborhoods, a representative community‐based sample of youths from this city (Jain et al., 2012). However, these studies typically span short periods—often only a few years—and focus primarily on adolescence, leaving the role of developmental assets in childhood in contributing to mental health in young adulthood largely unexplored. One exception is a previous study from our group using data from the Quebec Longitudinal Study of Child Development, in which we found that both internal and external assets at age 12 years were prospectively associated with less depressive symptoms and antisocial behaviors, as well as with more prosocial behaviors at age 20 years (Thibault et al., in press). Although this study shows preliminary evidence suggestive of long‐lasting contributions from developmental assets, it did not explore the role of timing. Understanding timing is essential for designing effective interventions, as identifying when experiences or exposures matter most allows interventions to be targeted at the stages of life when they can have the greatest impact (Smith et al., 2021; Zimmerman et al., 2013). This enables programs and policies to deliver specific interventions—such as social skills training in early childhood or peer‐support programs during adolescence—at the developmental stage when they are most likely to support mental health.

Life‐course and developmental theories posit that developmental outcomes are shaped by the timing of key life events and experiences (Ben‐Shlomo & Kuh, 2002; Elder, 1998). However, these models have primarily focused, both conceptually and empirically, on how the timing of adversity shapes development. Nevertheless, these frameworks may also inform research examining whether the timing of developmental assets is relevant to mental health in adulthood. For example, the Life Cycle Model of Stress (Lupien et al., 2009) posits that experiences may have distinct effects across development because brain regions involved in stress and emotion regulation mature and reorganize along different timelines across the life span, creating periods of relative heightened and reduced sensitivity to environmental influences. The prenatal period, early childhood, puberty, and old age are considered particularly sensitive due to increased neural plasticity (Takesian & Hensch, 2013). Mental health difficulties are also thought to commonly emerge in adolescence, when genetically programmed neural changes coincide with new social and environmental stressors (Andersen & Teicher, 2008). From this perspective, both adverse and promotive factors, such as developmental assets, may shape psychological functioning differently depending on when they occur, regardless of whether their effects are beneficial or harmful. For example, early childhood may represent a period in which external assets (e.g., social support, clear boundaries, and expectations from adults) are especially important for supporting foundational regulatory systems such as emotion regulation and stress responsivity, given young children's reliance on caregivers and proximal environments. In contrast, internal assets (e.g., social competencies, commitment to learning) may be particularly relevant during adolescence, a period characterized by heightened sensitivity to peer influence (Blakemore & Mills, 2014). Accordingly, considering the timing of developmental assets may help explain individual differences in adult mental health and clarify whether external and internal assets show different associations with these outcomes across development.

These theoretical models primarily support a sensitive period hypothesis, which posits that a factor, such as a developmental asset, exerts a particularly strong influence at specific ages or periods. Other key timing hypotheses investigated in longitudinal studies include the *accumulation* hypothesis, which suggests that the sustained exposure to positive factors over time promotes mental health, and the *recency* hypothesis, an extension of the accumulation hypothesis that emphasizes the stronger influence of more recent exposures (Smith et al., 2015; Smith et al., 2021). Consistent with the accumulation hypothesis, albeit largely in cross‐sectional research, the developmental asset literature suggests that a greater number of assets is associated with better mental health outcomes (Benson et al., 2011). However, accumulation effects have rarely been examined longitudinally or across developmental stages. Some longitudinal studies have focused on specific promotive factors. For example, trajectory‐based analyses of an ethnically diverse and representative sample from a southwestern county in the United States indicate that sustained prosocial behaviors across childhood and adolescence, a key indicator of social competencies, are associated with better mental health in emerging adulthood (Flynn et al., 2015).

Evidence relevant to the recency hypothesis remains limited but suggestive. In a longitudinal study of children from a diverse urban neighborhood in the United States, using a four‐wave cross‐lagged panel design spanning ages 10 and 30 years, social competencies were more strongly associated with subsequent internalizing problems at later developmental stages, consistent with a possible recency effect (Burt et al., 2008). However, the long intervals between assessments (e.g., ages 10 to 17 years, then 20 to 30 years) limit the ability to pinpoint when these effects are most pronounced. More broadly, commonly used longitudinal methods such as growth curve modeling or cross‐lagged approaches do not directly test competing life‐course hypotheses—such as sensitive period, accumulation, or recency models—across multiple assets. It remains unclear *which* and *when* promotive factors are most strongly associated with mental health in early adulthood.

Finally, most studies rely exclusively on self‐report measures to assess both developmental assets and mental health outcomes, introducing potential biases such as shared method variance, especially when assessments occur over short timeframes or concurrently. To limit these potential biases, the present study uses composite developmental asset scores evaluated by different informants (child, parent, teacher, interviewer) alongside self‐reported measures of mental health in emerging adulthood. In addition, research has disproportionately focused on mental health problems, such as anxiety and depression, while positive indicators of mental health are examined far less frequently. To provide a comprehensive view of mental health, the present study includes both positive (life satisfaction, happiness) and negative indicators (symptoms of anxiety and depression), reflecting their joint importance within a dual‐continua model of mental health (Keyes, 2005). Life satisfaction and happiness were examined separately, as they represent distinct dimensions of well‐being, with life satisfaction involving a cognitive, evaluative appraisal of one's life, and happiness capturing a more emotional, affective experience (Diener, 1984). Evidence shows that life satisfaction is more strongly associated with major life events and structural circumstances, such as marriage, childbirth, and income, underscoring the value of distinguishing between these complementary indicators (Kahneman & Deaton, 2010; Luhmann et al., 2012).

## OBJECTIVES AND HYPOTHESES OF THE PRESENT STUDY

The first objective of this study was to examine the associations between developmental assets measured from ages 5 to 17 years and mental health in emerging adulthood, specifically happiness, life satisfaction, anxiety symptoms, and depression symptoms between ages 19 and 22 years. We addressed this objective by examining (1) broad dimensions of internal and external assets and (2) specific asset categories individually, including four internal assets (commitment to learning, responsibility, social competencies, and positive identity) and four external assets (support, safety, boundaries and expectations, and constructive use of time). These asset categories were selected because necessary items were available longitudinally in this cohort and because they represent well‐established promotive factors frequently examined in childhood and adolescence (Bratman et al., 2021; Cairns et al., 2014; Magalhães, 2024; Zimmermann et al., 2020). The second objective was to determine which life‐course hypothesis—sensitive period, accumulation, and recency hypotheses—best explains these associations. We hypothesized that higher levels of developmental assets would be positively associated with greater well‐being and lower mental distress in emerging adulthood. Given limited prior evidence, we did not formulate specific hypotheses regarding whether the strength of these associations varies according to the type or timing of developmental assets, but we tested this possibility systematically.

## METHODS

### Sample and procedures

Participants were from the Quebec Longitudinal Study of Child Development (QLSCD), an ongoing population‐based cohort study conducted by the Institut de la statistique du Québec. The QLSCD recruited 2120 families with a child born between 1997 and 1998 in each administrative region of the Canadian province of Quebec, except those born in Northern Quebec, Cree Territory, Inuit Territory, and Native reserves (2.2% of all births). At its inception, the sample was representative of the population of Quebec and therefore consisted primarily of White, French‐speaking participants and included families from a wide range of socioeconomic backgrounds. Since 1998, participants have been evaluated annually or biannually. This study included 1344 participants (63% of the initial sample) for whom data on mental health outcomes were available between the ages of 19 and 22 years. Participants who remained involved in the study were more likely to be female and come from a higher socioeconomic background (see Missing Data). Developmental assets were evaluated across nine assessment waves between the ages of 5 and 17 years. Ethics committees of the Institut de la statistique du Québec approved each phase of the study. Parents provided written informed consent, and children provided assent at each data collection between 5 and 17 years. After the age of 18 years, participants provided written informed consent themselves. Details about the QLSCD are provided in the cohort profile (Orri et al., 2021) and online: https://www.jesuisjeserai.stat.gouv.qc.ca/. The secondary data analysis for this study was approved by the CHU Ste‐Justine and the University of Montreal ethics committee.

### Measures

#### Developmental assets from ages 5 to 17 years

We defined developmental assets based on the framework developed by the Search Institute and adapted by the Government of Yukon in collaboration with the Search Institute (Government of Yukon, 2020; Leffert et al., 1998). The Developmental Assets framework identifies 40 assets organized into two overarching dimensions: internal assets, which reflect individual strengths and values, and external assets, which capture supportive environments and social relationships. Each dimension comprises four categories: internal assets include commitment to learning, positive values, social competencies, and positive identity; external assets include support, empowering environments, boundaries and expectations, and constructive use of time. Although standardized measures of developmental assets exist, they were not available in the present longitudinal cohort. We therefore relied on the original definitions proposed by the Search Institute (Search Institute, 2005) to derive age‐sensitive asset indicators from available items. This approach allowed us to capture developmentally appropriate expressions of assets from childhood through late adolescence while leveraging the strengths of the longitudinal design. Although this limits direct comparability with studies using standardized instruments, it enhances the developmental relevance and ecological validity of the assets' measures across development and enables research questions that are rarely examined, such as the role of timing of developmental assets in adult mental health.

As described elsewhere (Thibault et al., in press), two research assistants (including coauthor MT) independently screened 40 questionnaires administered to the participating children, parents, and teachers across the nine waves of assessment between the ages of 5 (2003) and 17 years (2015). They screened approximately 20,000 items in search of items relevant to developmental assets. A total of 2162 items were identified as potentially relevant by the research assistants; 752 of those were selected by both research assistants and classified in the same asset category, providing inter‐rater agreement. The remaining 1410 items for which there was no inter‐rater agreement were examined by two expert researchers (coauthors AML and IOM). Of these, only 158 were retained. The final selection of 910 items (including the 752 for which inter‐rater agreement was found) was reviewed and validated by AML and IOM. Finally, 95 items were discarded as they were not discriminant enough, meaning that more than 80% of participants had the asset. The final number of items retained was 815.

Each item was dichotomized to indicate the presence (coded 1) or absence (coded 0) of a positive indicator relating to an asset, consistent with the count‐based approach of the Developmental Assets framework (Benson et al., 2011), which was also adopted in other studies (e.g., Zheng et al., 2022). For each item, asset presence was defined using a cutoff corresponding to the upper tertile (33%) of the item distribution. In prior sensitivity analyses (Thibault et al., in press), we showed that alternative thresholds (e.g., upper 40%) produced highly similar distributions of asset categories and dimensions. To create each asset, we summed up all the available items—provided that at least two‐thirds of the items were present for a given participant. This sum was then divided by the number of available items and multiplied by 10, resulting in a standardized score ranging from 0 to 10. A total of 23 assets were computed and grouped into eight asset categories, which were further organized into two overarching dimensions: Internal and External dimension scales (see descriptive statistics on Table 1). Examples of items used to compute asset categories under the Internal dimension were: (1) “*the child reads for pleasure*” for Commitment to Learning, (2) “*the child accepts responsibility for his or her actions*” for Responsibility, (3) “*the child is able to solve interpersonal conflicts*” for Social Competencies, (4) “*the child has self‐confidence*” for Positive Identity. For asset categories under the External dimension, examples of items were: (1) “*in the child's family, each person is accepted as they are*” for Support, (2) “*the child feels safe in their school*” for Safety, (3) “*it is important for the parent that their child gets good grades at school*” for Boundaries and Expectations, (4) “*the child frequently participates in artistic or sports activities*” for Constructive Use of Time. The number of items included in the Internal and External dimension scales was, respectively, 11 and 28 at age 5 years (2003), 45 and 54 at age 6 years (2004), 44 and 40 at age 7 years (2005), 51 and 47 at age 8 years (2006), 47 and 47 at age 10 years (2008), 48 and 45 at age 12 years (2010), 56 and 70 at age 13 years (2011), 36 and 56 at age 15 years (2013), 34 and 56 at age 17 years (2015) (Supplementary Materials; Table S1). We included all available items relevant to each asset at each age to maximize the information captured in our asset, category, and dimension indicators. At most time points, items were answered by three, sometimes four, different informants (child, mother, teacher, and interviewer), providing complementary perspectives (see Supplementary Materials, Table S1 for an account of the number of items answered by each informant). Again, because we relied on items collected within an existing cohort and prioritized developmentally appropriate indicators that vary in expression from ages 5 to 17, some assets could not be derived at certain ages (e.g., Responsibility at age 5 years; indicated by a dash in Table 1) or varied across developmental periods in terms of the number of items, item content, and informants. Although these differences reflect expected heterotypic heterogeneity across development, they may limit the direct comparability of assets over time and warrant caution in the interpretation of timing‐related findings. Future studies relying on different patterns of item availability, item content, and informants across development may provide complementary evidence regarding these timing effects, beyond variability attributable to measurement differences.

**TABLE 1:** Descriptive statistics for QLSCD participants with available data on mental health outcomes (*N* = 1344)^a^.

Potential confounding variables												
	5 months				17 months				29 months			
	*N*	%^b^	M (SD)	Range	*N*	%^b^	M (SD)	Range	*N*	%^b^	M (SD)	Range
Sex (0 = M; 1 = F)	1344	57%	–	–	–	–	–	–	–	–	–	–
Canadian origin of the mother (0 = no; 1 = yes)	1335	65%	–	–	–	–	–	–	–	–	–	–
Canadian origin of father (0 = no; 1 = yes)	1244	68%	–	–	–	–	–	–	–	–	–	–
Family type (0 = single; 1 = two‐parent)	1340	94%	–	–	1336	92%	–	–	1334	90%	–	–
Socioeconomic status	1338	–	.12 (.98)	−3.01 to 2.84	1338	.10 (.99)	.10 (.99)	−3.01 to 2.85	1332	.10 (.99)	.09 (1.00)	−2.83 to 5.73
Children's hyperactivity	–	–	–	–	1339	–	3.45 (2.16)	0–10	1337	–	3.83 (2.33)	0–10
Children's aggression	–	–	–	–	1339	–	1.30 (1.25)	0–7.92	1337	–	1.71 (1.38)	0–10
Children's emotional problems	–	–	–	–	1339	–	.86 (1.00)	0–7.86	1337	–	.48 (.92)	0–6.67

Developmental Asset Dimensions and Categories^c^																		
	5 years (2003)		6 years (2004)		7 years (2005)		8 years (2006)		10 years (2008)		12 years (2010)		13 years (2011)		15 years (2013)		17 years (2015)	
	*N*	M (SD)	*N*	M (SD)	*N*	M (SD)	*N*	M (SD)	*N*	M (SD)	*N*	M (SD)	*N*	M (SD)	*N*	M (SD)	*N*	M (SD)
Internal dimension	1241	4.28 (2.21)	755	5.31 (1.78)	1015	4.58 (2.01)	996	4.58 (1.83)	798	4.60 (1.87)	824	4.48 (1.90)	1024	4.19 (1.95)	1196	3.75 (1.71)	1115	3.82 (1.60)
Commitment to learning	1241	6.34 (3.33)	687	5.60 (1.91)	1133	4.28 (2.13)	991	4.64 (2.05)	797	4.05 (2.12)	1108	3.70 (2.20)	1024	3.70 (2.24)	1196	2.99 (2.09)	1009	3.02 (2.02)
Responsibility	–	–	765	7.42 (4.38)	1013	4.13 (4.93)	1005	4.52 (4.98)	804	4.99 (5.00)	832	5.30 (4.99)	890	2.80 (4.49)	1195	2.74 (3.96)	1115	3.03 (4.16)
Social competencies	1248	3.10 (2.74)	767	5.26 (2.15)	997	4.84 (2.58)	1006	4.54 (2.13)	785	4.96 (2.07)	824	5.01 (2.12)	1029	4.45 (2.28)	1195	4.45 (2.05)	1113	4.56 (2.02)
Positive identity	–	–	703	3.15 (2.42)	1009	4.24 (3.39)	973	4.61 (3.35)	805	2.29 (4.20)	1108	5.81 (2.72)	1025	5.14 (2.78)	–	–	–	–
External dimension	1248	2.21 (1.39)	1060	3.96 (1.21)	1142	4.13 (1.28)	997	3.72 (1.13)	1063	4.22 (1.39)	1088	4.45 (1.52)	882	4.72 (1.61)	970	4.27 (1.71)	867	3.89 (1.76)
Support	1248	2.22 (2.06)	1051	4.53 (1.50)	1142	5.36 (1.66)	998	4.34 (1.49)	1017	4.92 (1.87)	1059	4.83 (1.81)	958	4.73 (181)	971	4.18 (1.96)	870	3.77 (1.99)
Safety	–	–	–	–	–	–	–	–	–	–	1109	5.23 (5.00)	654	4.42 (3.60)	1194	3.60 (4.80)	1101	4.46 (4.97)
Boundaries and expectations	1238	2.43 (2.53)	732	3.81 (1.49)	1018	2.83 (2.44)	960	3.85 (1.91)	1015	4.37 (1.95)	1133	4.22 (1.98)	1026	4.92 (2.22)	975	5.09 (2.03)	902	4.54 (2.09)
Constructive use of time	1248	2.00 (1.54)	1146	2.56 (1.46)	1047	2.11 (1.66)	995	2.45 (1.55)	1081	2.72 (1.95)	863	2.28 (2.41)	788	2.94 (2.56)	866	2.94 (2.56)	722	2.80 (2.24)

Mental health outcomes												
	19 years (2017)			20 years (2018)			21 years (2019)			22 years (2020)		
	*N*	M (SD)	Range	*N*	M (SD)	Range	*N*	M (SD)	Range	*N*	M (SD)	Range
Life satisfaction	1271	7.50 (1.90)	0–10	–	–	–	1241	7.27 (1.83)	0–10	–	–	–
Happiness	1271	7.49 (1.86)	0–10	–	–	–	1241	7.25 (1.93)	0–10	–	–	–
Anxiety symptoms	–	–	–	1220	4.67 (4.63)	0–21	–	–	–	1164	4.54 (4.79)	0–21
Depression symptoms	–	–	–	1221	9.25 (6.44)	0–36	–	–	–	1162	9.67 (6.83)	0–36

Abbreviations: M, mean; *N*, number of participants; QLSCD, Quebec Longitudinal Study of Child Development; SD, standard deviation.

^a^ Data were compiled from the final master file of the Quebec Longitudinal Study of Child Development (1998–2018), ©Gouvernement du Québec, Institut de la statistique du Québec.

^b^ Percentages of values = 1.

^c^ Scores for developmental assets ranged from 0 to 10. The number of participants varied across time points because of attrition and some items were not discriminatory enough and had to be discarded (i.e., when most participants answered positively).

In a previous study, we examined the psychometric properties of asset scales at ages 12, 13, 15, and 17 years (Thibault et al., in press). Most asset scales demonstrated adequate internal consistency. A few scales showed lower inter‐item correlations, possibly reflecting the use of multiple informants and observations drawn from distinct contexts. The multi‐factorial structure of the scales, incorporating all asset categories within a single model, was not tested in this study. This decision was guided by a theory‐driven approach, in which we relied on the existing measurement framework to conceptualize developmental assets. Overall, the asset dimensions showed adequate predictive validity, as they were associated with behavioral and emotional problems in early adulthood and moderate stability over time. We repeated the validation analyses for the asset scales measured at ages 5, 6, 7, 8, and 10 years, yielding similar results (Supplementary Materials).

#### Mental health outcomes from ages 19 to 22 years

Mental health outcomes were evaluated using self‐reports between the ages of 19 (2017) and 22 years (2020). To provide a comprehensive view of mental health during emerging adulthood, we included indicators of both positive and negative mental health.

##### Life satisfaction and happiness

Participants reported on their life satisfaction and happiness on a 0–10 scale using questions inspired by the Canadian Community Health Survey (Statistics Canada, 2015) at ages 19 (2017) and 21 years (2019). At each time point, participants answered two questions, one relating to life satisfaction and the other to happiness. They reported on their overall feelings about their life from very unsatisfied (score 0) to very satisfied (score 10) and on their level of happiness from very unhappy (score 0) to very happy (score 10). Because life satisfaction and happiness showed moderate stability between ages 19 and 21 years (*r*_life satisfaction_ = .51; *r*_happiness_ = .49), scores were averaged across the two time points for each construct. Although this approach may obscure within‐person change, it allowed for reducing the number of tests examining moderately stable outcomes.

##### Anxiety and depression symptoms

Participants reported on their anxiety and depression symptoms at ages 20 (2018) and 22 years (2020). Anxiety symptoms were evaluated using the Generalized Anxiety Disorder 7‐item (GAD‐7) (Spitzer et al., 2006). Participants had to answer seven questions about how often they had been bothered by feelings of anxiety, worry, irritation, tenseness, and fear during the past two weeks on a 0–3 scale ranging from “never” to “almost every day”. A mean score was computed for participants who had at least 5 out of 7 available items and multiplied by the total number of available items (range = 0 to 21). Depression symptoms were evaluated using the Center for Epidemiologic Studies Depression Scale (CES‐D) (Radloff, 1977). Participants had to answer 13 questions about their feelings and behaviors during the past week on a 0–3 scale ranging from “rarely or never” to “most of the time or all the time”. The mean score was computed for participants who had at least 10 out of 13 items and multiplied by the total number of items (range = 0 to 39). Because anxiety and depression symptoms were moderately stable from age 20 to 22 years (*r*_anxiety_ = .49; *r*_depression_ = .52), we computed their average across the two time points to limit the number of statistical tests.

#### Covariates measured in early childhood

The main analyses adjusted for the following sociodemographic and behavioral outcomes during early childhood: (1) biological sex reported at 5 months, (2) socioeconomic status evaluated at 5, 17, and 29 months using a composite obtained from a factorial analysis measuring parental educational level, parental occupation, and annual gross income (Willms & Shields, 1996), (3) origin of the parents at 5 months (0 = not Canadian; 1 = Canadian), (4) family type at 5, 17, and 29 months (0 = single‐parent family status at least once across the three time points; 1 = two‐parent family status at all three time points), (5) externalizing and (6) internalizing behaviors evaluated by the mother at 17 and 29 months. Mothers evaluated their child's externalizing and internalizing behaviors by answering questions on a 3‐point scale (1 = *never or not true*; 2 = *sometimes or somewhat true*; 3 = *often or very true*). Externalizing behaviors were measured using 7 items evaluating hyperactivity (e.g., *cannot stay in place, is agitated*) and 12 items evaluating physical aggression (e.g., *kicks other children with his or her feet*) at 17 months. At 29 months, hyperactivity and aggression were evaluated using 5 and 13 items similar to those used at 17 months. Hyperactivity and aggression scores at 17 and 29 months were standardized on a 0‐to‐10 scale. We then calculated the average across hyperactivity and aggression scores at 17 and 29 months, as evidence suggests that these behaviors cluster under a global externalizing behavior construct in early childhood (Michelini et al., 2023). We did the same for internalizing behaviors, which were measured using 7 and 3 items assessing emotional problems at 17 and 29 months, respectively (e.g., *not as happy as other children*), evaluated by mothers on the same 3‐point scale. These composite scores were included to statistically control for stable, pre‐existent individual differences in emotional and behavioral problems when examining associations between developmental assets and adult mental health outcomes.

### Missing data

This study included a subsample of 1344 participants (63.4%) with complete data on mental health outcomes at ages 19, 20, 21, or 22 years, drawn from the original 2120 participants enrolled in the QLSCD at 5 months. Using chi‐square tests and *t*‐tests, we examined potential differences in sociodemographic and behavioral indicators between the included (*n* = 1344) and excluded (*n* = 776) participants. Compared to those not included, participants retained in the study were more likely to be female [*χ* ^2^(df, *N* = 2120) = 93.44, *p* < .001], to have grown up in a two‐parent family household [*χ* ^2^(df, *N* = 2001) = 93.44, *p* < .01], and to have a Canadian‐born father [*χ* ^2^(df, *N* = 1929) = 9.17, *p* < .01], as well as to come from a higher socioeconomic background [*t*(1,589.10) = −7.46, p < .01]. No significant differences were observed in maternal country of origin [*χ* ^2^(df, *N* = 2105) = 2.17, *p* = .14], or in early childhood levels of externalizing [*t*(1,348.90) = 1.79, *p* = .07] and internalizing behaviors [*t*(1,316.50) = 1.00, *p* = .32]. To reduce potential bias associated with non‐random patterns of missingness, these variables were included as covariates in the main analyses, although residual bias due to selective attrition cannot be ruled out. Missing data on developmental assets and confounders were imputed using multiple chain equations, creating four sets of 20 imputed datasets (mice; version 3.16.0) in R (version 4.2.3), as described in the Supplementary Materials (Buuren & Groothuis‐Oudshoorn, 2011; R Core Team, 2024).

### Analyses

Descriptive statistics, including the number of participants at each assessment wave, frequencies (for binary variables), mean and standard deviation estimates (for continuous variables), and value ranges for all variables, are presented in Table 1.

To examine the role of timing in the association between developmental assets and young adults' mental health, we first ran multiple regressions and then a two‐stage structured life course modeling approach (SLCMA) (Mishra et al., 2009; Smith et al., 2015; Smith et al., 2021). In both steps (multiple regressions and the SLCMA), we examined whether developmental assets were associated with each of the four mental health outcomes in emerging adulthood (life satisfaction, happiness, anxiety, and depression symptoms). In the multiple regression models and SLCMA, we included covariates selected because of their associations with developmental assets or mental health outcomes: sex assigned at birth, origin of the parents, socioeconomic status, family type, and internalizing and externalizing problems in early childhood. In the multiple regression models, covariates were entered as independent variables. In the SLCMA, we used the Frisch‐Waugh‐Lovell theorem, where we regressed both exposures and outcomes on covariates. Analyses were conducted in R version 4.2.3 (R Core Team, 2024) using the packages lars version 1.3 (Hastie & Efron, 2022) and selective Inference version 1.2.5 (Tibshirani et al., 2019).

#### Multiple regression models

We first examined whether developmental assets were significantly associated with mental health outcomes by conducting multiple regression models using the imputed QLSCD data. For each of the four outcomes, we ran models including assets measured at each of the nine time points (ages 5, 6, 7, 8, 10, 12, 13, 15, 17 years), for a total of 36 models. We accounted for these 36 tests using the Benjamini‐Hochberg false‐discovery rate (FDR) correction to limit the probability of making Type I errors (i.e., rejecting a true null hypothesis). These models included simultaneously the internal and external dimension scales to assess their unique association with the outcome. We then reran these analyses according to the eight categories scales simultaneously (commitment to learning, responsibility, social competencies, positive identity, support, safety, boundaries and expectations, and constructive use of time), again for 36 models (nine time points*four outcomes). We adjusted the threshold of significance for p‐values using the Benjamini‐Hochberg FDR correction. Based on these results, we retained only the developmental assets that showed at least one significant association with a mental health outcome for testing the life‐course hypotheses. Asset dimensions or categories that did not meet this criterion were excluded from subsequent analyses to limit the number of tests. We acknowledge that this pre‐screening step introduces an additional layer of selection, such that subsequent inferences are conditional on both the initial filtering process and the SLCMA procedure itself. This should therefore be taken into consideration when interpreting effect estimates and their associated uncertainty.

#### Structured Life‐Course Modeling Approach (SLCMA)

We then used the SLCMA to examine the role of timing, using asset dimensions or categories that showed significant associations with the mental health outcomes in the multiple regression models. The SLCMA is a two‐stage approach designed to compare competing life‐course hypotheses concurrently, optimizing the most predictive model while maintaining statistical power (Smith et al., 2021). In stage 1, the SLCMA uses Least Angle Regression (LARS) to identify the life‐course hypothesis (or set of hypotheses) that explains the most outcome variance. LARS is a stepwise method that gradually adds predictors to a model by selecting those that explain the greatest variance in the outcome (Efron et al., 2004). Unlike ordinary least squares (OLS) regression, which fits a single model using a pre‐selected set of predictors, LARS evaluates alternative models to determine which predictors should enter the model and in what sequence. LARS is particularly suited for examining many correlated predictors, such as when comparing different life‐course hypotheses that rely on the same exposure variables weighted differently. However, because LARS selects variables based on the observed data, it is susceptible to selection bias. In stage 2, the SLCMA applies a post‐selection inference procedure to obtain unbiased estimates of the final model parameters, including coefficients, confidence intervals, and *p*‐values, by accounting for the model selection process conducted in stage 1. This procedure conditions on the event that a specific set of variables was selected by the LARS algorithm, thereby appropriately reflecting the uncertainty introduced by data‐driven variable selection (Lee et al., 2016). It is important to note that the post‐selection inference preserves the directional interpretation of regression coefficients, while all uncertainty statements are conditional on the selection process. In contrast, global fit measures such as *R* ^2^ are not selection‐adjusted and therefore should not be interpreted in the same inferential manner as in ordinary OLS regression; however, they remain descriptively analogous in that they summarize the proportion of outcome variance explained by the selected model.

In the first stage, we generated three life‐course hypotheses: accumulation (coded as the total number of developmental assets experienced, i.e., 0, 1, 2, 3, 4, 5, etc.), sensitive period (coded as 1 if a developmental asset was experienced at a particular age and coded as 0 otherwise), and recency (which weighted more recent exposures more heavily than earlier ones, based on their temporal proximity to the mental health outcomes). The three life‐course models were entered together into a LARS model. Using the Frisch‐Waugh‐Lovell theorem, we regressed both exposures and outcomes on covariates (sex assigned at birth, origin of the parents, socioeconomic status, family type, and internalizing and externalizing problems in early childhood) before running LARS. For each LARS model, an elbow plot modeling the *R* ^2^ values for each increasingly complex model was created. We retained the number of hypotheses coinciding with where the elbow occurred (highest *R* ^2^). When elbow plots in stage 1 suggested more than one selected hypothesis, we examined the *p*‐values for each hypothesis in stage 2 (post‐inference). We retained more than one hypothesis only when post‐inference *p*‐values were both < .05.

#### Sensitivity analyses

We subsequently assessed whether the results were robust by examining the timing hypotheses according to each developmental period rather than specific ages. To do so, we repeated the SLCMA described previously, this time combining the dimension or category asset scales into three periods: “early childhood”: ages 5, 6, and 7 years; “late childhood”: ages 8, 10, and 12 years; and “adolescence”: ages 13, 15, and 17 years.

## RESULTS

Descriptive statistics for the main variables are presented in Table 1. The distribution of the life satisfaction and happiness 0‐to‐10 scales (averaged across ages 19 and 21 years) was both negatively skewed, whereby most participants reported scores that were higher than 5 (Statistics Canada, 2015). Nonetheless, 20% and 22% scored 6 or below on life satisfaction and happiness, respectively, reflecting a subset of participants with comparatively lower well‐being. For anxiety and depression (averaged across ages 20 and 22 years), 13% scored 10 or higher on the GAD‐7 (0‐to‐21; Spitzer et al., 2006) and 42% scored 9.6 or higher on the CES‐D (0‐to‐39; the cutoff was determined proportionally to the original 20‐item CES‐D, for which the cutoff is 16/60; Radloff, 1977). Therefore, more than 1 in 10 participants had clinically relevant anxiety symptoms, and more than 1 in 3 had clinically relevant depression symptoms over the same period. We provide correlations between outcome variables in the Supplementary Materials (Table S2). Because life satisfaction and happiness were strongly correlated at ages 19 and 21 years (*r* = .79–.81), and yielded similar results across all analyses, we present findings for life satisfaction only. Results for happiness are provided as sensitivity analyses in the Supplementary Materials. Notably, all significant associations were generally small in magnitude, with most standardized estimates below *β* = .20 across outcomes (Cohen, 1988).

### Multiple regression models to examine associations between developmental assets and mental health outcomes

**FIGURE 1:**
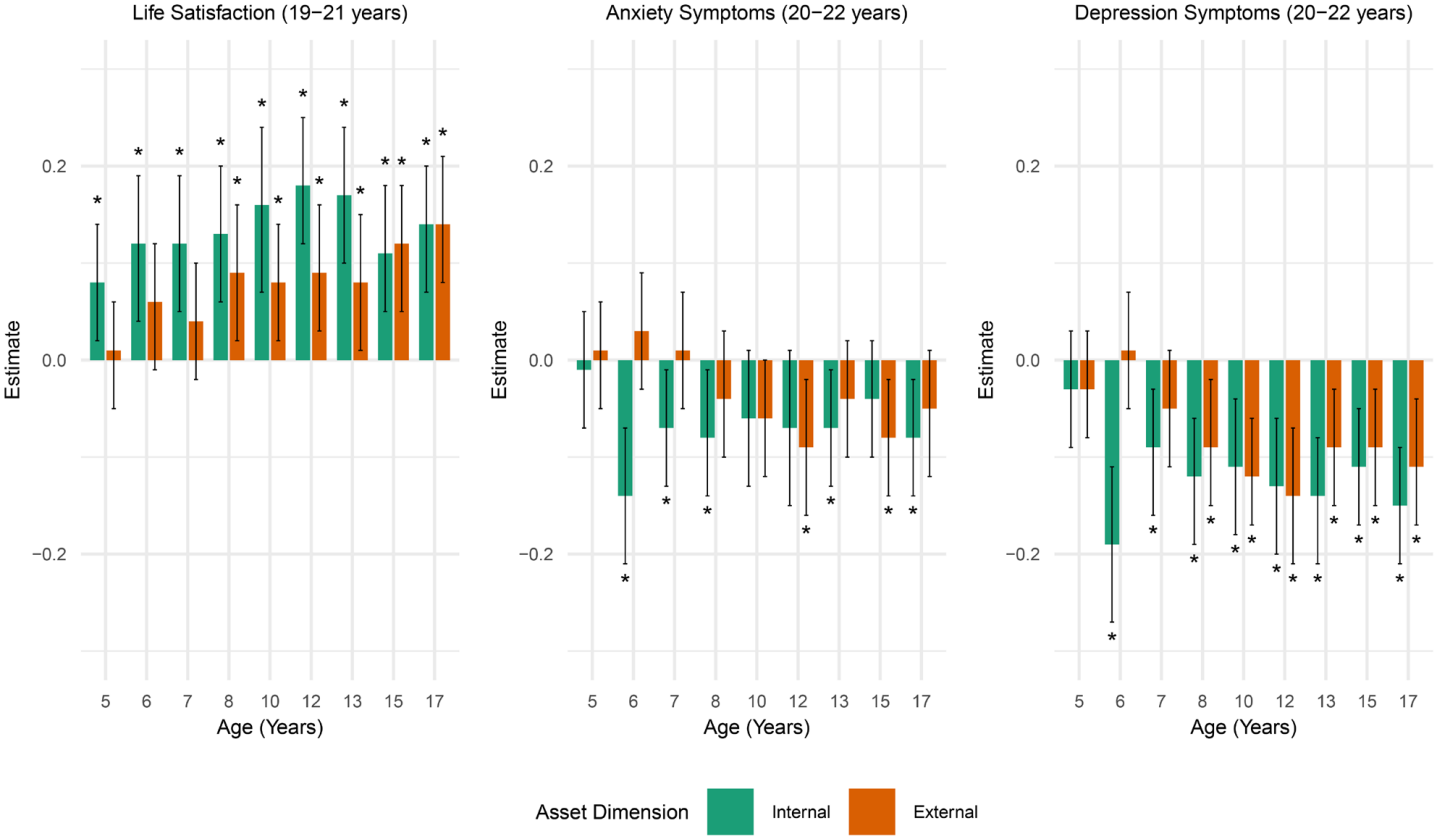
Multiple Regression Models Predicting Mental Health Outcomes from the Internal and External Dimension Scales in the QLSCD^a^. Internal and External dimension scales were entered together in the same model; their estimates are thus independent. Models were adjusted for covariates (sex, socioeconomic status, parental origin, family type, externalizing and internalizing problems in early childhood). Continuous predictors and the outcome were z‐standardized prior to analysis; regression coefficients, therefore, represent standardized estimates. QLSCD, Quebec Longitudinal Study of Child Development. ^a^Data were compiled from the final master file of the Quebec Longitudinal Study of Child Development (1998–2018), ©Gouvernement du Québec, Institut de la statistique du Québec. *p < .05 after false‐discovery rate correction.

We first examined whether developmental assets were associated with mental health outcomes using multiple regression models. Figure 1 presents the results of the regression models accounting for a wide range of family‐ and child‐level confounding variables (see the Supplementary Materials for the estimates; Table S3). Across nearly all assessment time points, both internal and external asset dimensions were uniquely and independently associated with all outcomes, showing positive associations with life satisfaction and negative associations with anxiety and depression symptoms.

**FIGURE 2:**
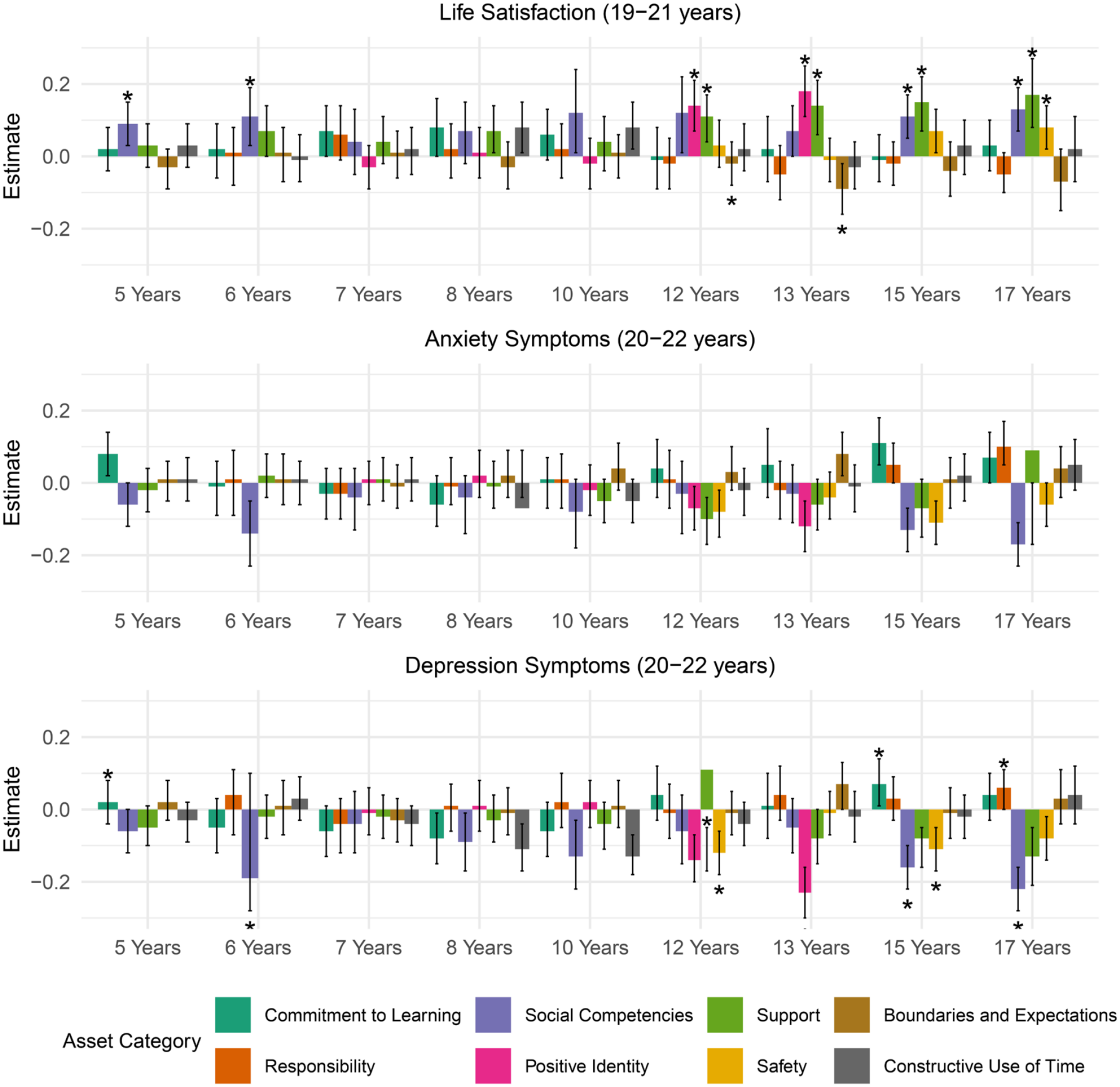
Multiple Regression Models Predicting Mental Health Outcomes from Each Asset Categories in the QLSCD^a^. All eight asset categories were entered together in the same model; their estimates are thus independent. Models were also adjusted for covariates (sex, socioeconomic status, parental origin, family type, externalizing and internalizing problems in early childhood). Continuous predictors and the outcome were z‐standardized prior to analysis; regression coefficients, therefore, represent standardized estimates. QLSCD, Quebec Longitudinal Study of Child Development. ^a^Data were compiled from the final master file of the Quebec Longitudinal Study of Child Development (1998–2018), ©Gouvernement du Québec, Institut de la statistique du Québec. *p < .05 after false‐discovery rate correction.

Associations between the eight individual asset categories and mental health outcomes obtained via multiple regression models are presented in Figure 2 (see the Supplementary Materials for the estimates; Table S4). Unlike the internal and external dimensions, which largely showed unique and independent associations with the outcomes, only a few individual asset categories were significantly and independently associated with the outcomes when included together in the same model. Social Competencies (internal asset), Positive Identity (internal asset), and Support (external asset) showed significant associations with all outcomes, whereas Constructive Use of Time (external asset), Safety (external asset), Boundaries and Expectations (external asset), Commitment to Learning (internal asset) and Responsibility (internal asset) only showed a few significant associations with one or two outcomes. Asset categories that did not show unique associations with specific outcomes (e.g., Commitment to Learning with life satisfaction) were excluded from subsequent SLCMA analyses for these outcomes.

### Structured life‐course modeling approach (SLCMA) to examine timing hypotheses

We then examined which timing hypotheses best accounted for the associations between developmental assets and mental health outcomes using the SLCMA.

#### Dimension scales

Recency emerged as the selected life‐course hypothesis reflecting the association between both internal and external asset dimensions and life satisfaction, and between the internal asset dimension and depression symptoms (Table 2). In contrast, the sensitive period hypothesis was selected for the associations between the external dimension and symptoms of anxiety and depression, where external assets at age 12 years specifically showed the strongest associations. For the association between internal assets and anxiety symptoms, estimates from the post‐selection inference were not statistically significant.

**TABLE 2:** SLCMA‐selected life‐course hypothesis for internal and external dimension scales by mental health outcome.

	Life satisfaction 19–21 years			Anxiety symptoms 20–22 years			Depression symptoms 20–22 years		
	Step 1		Step 2	Step 1		Step 2	Step 1		Step 2
	Model	*R* ^2^	Estimate (95% CI)	Model	*R* ^2^	Estimate (95% CI)	Model	*R* ^2^	Estimate (95% CI)
Internal assets	Recency	.062	.**002 (.002, .003)**	6 years	.001	−.049 (−.085, .004)	Recency	.041	**−.002 (−.003, −.002)**
External assets	Recency	.017	.**002 (.001, .003)**	12 years	.009	**−.080 (−.114, −.039)**	12 years	.005	**−.126 (−.159, −.005)**

*Note*: Step 1 corresponds to the Least Angle Regression (LARS) models. Step 2 corresponds to the post‐selection inference. Estimates in bold were significant according to a Benjamini‐Hochberg FDR‐adjusted *p‐*value < .05 considering the six models. *R* ^2^ represents the proportion of variance in mental health outcomes explained by the developmental assets, according to the selected hypothesis and after adjusting for covariates (sex, socioeconomic status, parental origin, family type, externalizing and internalizing problems in early childhood). For the recency hypothesis, the estimate from step 2 represents the change in the mental health outcome *z*‐score for a one‐point increase in exposure, weighted by the temporal proximity of exposure to the outcome. For the time‐specific, sensitive period hypothesis, the estimate reflects the difference in the outcome *z*‐score between individuals exposed vs. unexposed at this specific age. Data were compiled from the final master file of the Quebec Longitudinal Study of Child Development (1998–2018), ©Gouvernement du Québec, Institut de la statistique du Québec.

Abbreviations: CI, confidence intervals; SLCMA, Structured Life‐course Modeling Approach.

#### Category scales

We then examined the timing hypotheses of each asset category individually. Overall, results were partly consistent with and complementary to those noted for the broader dimension scales. Results are presented in Table 3.

**TABLE 3:** SLCMA‐selected life‐course hypothesis for each internal and external asset category by mental health outcome.

	Life satisfaction 19–21 years			Anxiety symptoms 20–22 years			Depression symptoms 20–22 years		
	Step 1		Step 2	Step 1		Step 2	Step 1		Step 2
	Model	*R* ^2^	Estimate (95% CI)	Model	*R* ^2^	Estimate (95% CI)	Model	*R* ^2^	Estimate (95% CI)
**Internal assets**									
Commitment to learning	–	–	–	8 years	.001	−.040 (−.065, .023)	–	–	–
	–	–	–	5 years	<.001	.025 (−.015, .041)	–	–	–
Responsibility	–	–	–	17 years	.006	.**025 (.005, .038)**	–	–	–
Social competencies	Recency	.057	.**002 (.002, .003)**	17 years	.006	**−.087 (−.111, −.017)**	Recency	.013	**−.002 (−.003, −.001)**
Positive identity	13 years	.026	.**069 (.049, .089)**	13 years	.006	**−.044 (−.063, −.004)**	13 years	.039	**−.075 (−.095, −.055)**
	12 years	.070	.**047 (.026, .067)**				12 years	.077	**−.043 (−.064, −.023)**
**External assets**									
Support	Recency	.030	.**002 (.001, .002)**	12 years	.012	**−.069 (−.098, −.037)**	Recency	.003	−.001 (−.002, .001)
Safety	Recency	.023	.**001 (.000, .001)**	Accumulation	.010	**−.011 (−.016, −.005)**	Accumulation	.011	**−.011 (−.018, −.005)**
							12 years	.040	**−.020 (−.033, −.005)**
Boundaries and expectations	Recency	.002	.000 (−.000, .001)	–	–	–	–	–	–
Constructive use of time	–	–	–	–	–	–	10 years	.010	**−.069 (−.095, −.005)**

*Note*: Dashes indicate SLCMA models that were not tested because multiple regression results showed no significant association between the asset category and the outcome. Step 1 corresponds to the Least Angle Regression (LARS) models. Step 2 corresponds to the post‐selection inference. Estimates in bold were significant according to a Benjamini‐Hochberg FDR‐adjusted *p‐*value < .05 considering the 16 models. *R* ^2^ represents the proportion of variance in mental health outcomes explained by the developmental assets, according to the selected hypothesis and after adjusting for covariates (sex, socioeconomic status, parental origin, family type, externalizing and internalizing problems in early childhood). For the recency hypothesis, the estimate from step 2 represents the change in the mental health outcome *z*‐score for a one‐point increase in exposure, weighted by the temporal proximity of exposure to the outcome. For the time‐specific, sensitive period hypothesis, the estimate reflects the difference in the outcome *z*‐score between individuals exposed vs. unexposed at this specific age. Data were compiled from the final master file of the Quebec Longitudinal Study of Child Development (1998–2018), ©Gouvernement du Québec, Institut de la statistique du Québec.

Abbreviations: CI, confidence intervals; SLCMA, Structured Life‐course Modeling Approach.

When predicting life satisfaction, findings were mostly consistent with those reported at the dimension level, which favored the recency hypothesis. The recency hypothesis emerged as the selected model for Social Competencies (internal asset), Support (external asset), and Safety (external asset) in relation to life satisfaction. However, findings regarding Positive Identity (internal asset) were inconsistent with previous results on the broad internal asset dimension. The sensitive period was selected rather than the recency hypothesis, indicating that Positive Identity at ages 12 and 13 years showed the strongest association with life satisfaction.

For anxiety symptoms, consistent with our findings for the broader asset dimensions, the sensitive period at age 12 years was selected for the association between Support and anxiety symptoms. In contrast, although no timing hypothesis significantly accounted for the association between the overall internal assets and anxiety symptoms, specific internal asset categories showed significant timing effects: the sensitive period hypothesis was selected for Responsibility and Social Competencies at age 17 years, and for Positive Identity at age 13 years. Although the external dimension at age 12 years explained the most variance in anxiety symptoms, the association between Safety and anxiety symptoms was better captured by the accumulation hypothesis.

Finally, for depression symptoms, consistent with findings for the overall internal dimension, the recency hypothesis was selected for the association between Social Competencies and depression symptoms. In contrast, Positive Identity at ages 12 and 13 years was most strongly associated with depression symptoms, in line with the sensitive period hypothesis, despite the recency effect observed for the broader internal dimension. Similarly, although the external dimension at age 12 years explained the most variance in depression symptoms, the association between Safety and depression symptoms was best captured by the accumulation hypothesis, and a secondary hypothesis was provided by the sensitive period at age 12 years.

### Sensitivity analyses to examine developmental periods rather than specific ages

We tested the robustness of the results by repeating all analyses using broader developmental periods—early childhood (ages 5–7 years), late childhood (8–12 years), and adolescence (13–17 years)—instead of specific ages. Results are reported in the Supplementary Materials (Table S5–S8; Figures S1 and S2). For both Internal and External dimension scales, results closely mirrored those obtained with age‐specific assets, with the recency hypothesis selected for most outcomes. Late childhood remained the most predictive period for the association between External assets and both anxiety and depression symptoms (previously at age 12 years). The only difference was that Internal assets were now associated with anxiety symptoms under the recency hypothesis, whereas this association was not initially significant. This change may reflect the broader timeframes combining assets measured at different ages.

We conducted the same analyses for asset categories using broader developmental periods. Because Positive Identity was measured only from ages 6 to 13 years and Safety only from ages 12 to 17 years, sensitivity analyses were not performed for these two categories. Fewer associations reached significance compared with the age‐specific analyses. Overall, findings based on broader developmental periods were consistent with the age‐specific analyses, in which stronger associations emerged at specific ages or periods, or following a recency pattern rather than an accumulation pattern, reinforcing the robustness of the main results.

## DISCUSSION

The present study aimed to examine which developmental assets contribute to shaping mental health outcomes in emerging adulthood, and whether the timing of exposure to, or possession of, these assets across childhood and adolescence influenced these outcomes. Three key findings emerged from this study: (1) among the competing life‐course hypotheses, the sensitive period hypothesis was frequently selected as capturing the strongest associations between developmental assets and negative outcomes (i.e., anxiety and depression symptoms), with key periods corresponding to a major developmental milestone in this cohort: the year preceding the transition from primary to secondary school in this cohort (age 12 years); (2) the recency hypothesis explained the greatest proportion of variance in the positive outcomes of life satisfaction and happiness for both internal and external assets; and (3) analyses conducted at the level of individual asset categories raised the possibility that the strength of the associations between developmental assets and functioning in early adulthood may vary across childhood and adolescence depending on the nature of the asset categories considered.

However, these findings should be interpreted as preliminary for three main reasons. First, a regression‐based screening step was conducted before the main SLCMA analyses, such that inferences were conditional on both this initial filtering process and the subsequent LARS selection procedure. Second, developmental asset indicators were not identical across the eight time points spanning 11 years (i.e., ages 5–17), as items were drawn from developmentally appropriate questionnaires at each time point. Third, all observed effect sizes were small, indicating that a substantial proportion of variance in mental health outcomes remains unexplained. This likely reflects the contribution of genetic and biological factors not readily captured in the individual assets, along with their interplay with environmental influences both within and beyond the external assets examined in the present study (Uher & Zwicker, 2017). Although developmental assets represent only a significant but small part of the broader societal, cultural, and individual factors part of the etiological framework, the present study extends current understanding by systematically examining when these influences may matter most for mental health outcomes in emerging adulthood. Such effect sizes are typical in longitudinal research, where long time intervals between the measurements of predictors and outcomes often attenuate associations. Nonetheless, given the rising prevalence rates of anxiety and depression (Cybulski et al., 2021; Dykxhoorn et al., 2024; McGorry et al., 2024; Twenge et al., 2019), even modest improvements in promotive factors may translate into meaningful gains in life satisfaction and reductions in anxiety and depressive symptoms for many young adults. Findings nevertheless invite future studies to examine further timing‐related patterns of association between developmental assets and psychosocial functioning in early adulthood.

### Developmental assets at age 12: A sensitive period for adult anxiety and depression symptoms

External assets present during the year preceding the transition to secondary school (age 12) were more strongly associated with anxiety and depression symptoms in emerging adulthood (ages 20–22), although the observed effect sizes were small. Among the life‐course hypotheses tested, the sensitive period hypothesis centered around age 12 was selected as the most strongly related to these outcomes. Importantly, this finding should not be interpreted as evidence of a sensitive period in a causal or biological sense, but rather as indicating that support, safety, boundaries, and expectations from adults, and constructive use of time were collectively more strongly associated with anxiety and depression symptoms in early adulthood when present during that period. Together, these assets reflect supportive, structured, and health‐promotive social environments that may foster autonomy and positive identity formation. Exposure to such environments during major educational and social transitions may help young people navigate the challenges of early adolescence, potentially conferring enduring benefits for mental health (Bharara, 2020).

The importance of this timing effect may be understood in light of both contextual and developmental processes. The transition to secondary school coincides with heightened academic, social, and environmental demands, making youth more sensitive to variations in external support and structure. This period also overlaps with puberty, which the Life Cycle Model of Stress identifies as a window of increased neurobiological plasticity (Lupien et al., 2009). During this stage, stress‐sensitive brain systems involved in emotional processing and regulation continue to mature, rendering adolescents more vulnerable to environmental influences. Consistent with this perspective, early adolescence has also been described as a sensitive period for social processing, during which youth become especially attuned to social cues, evaluation, and safety within their environments (Blakemore & Mills, 2014).

Our findings align with and extend prior research showing the protective role of parental and peer support, school connectedness, and perceived safety during the transition from primary to secondary school among adolescents from Western Australia (Lester & Cross, 2015; Waters et al., 2014). By showing that these external assets predict mental health outcomes nearly a decade later, our study underscores the long‐term importance of supportive and structured environments during early adolescence. It also highlights the potential value of prevention strategies targeting the transition to secondary school, particularly those that strengthen positive parental behaviors (support, healthy boundaries, and expectations), promote open family communication, and encourage school involvement in reducing anxiety and depression symptomatology in early adulthood.

### Later assets are more strongly associated with life satisfaction and depression symptoms: Evidence from the Recency hypothesis

By comparison, life satisfaction was consistently best explained by the recency hypothesis for both internal and external assets (although effect sizes were small). This suggests that as assets are acquired or experienced later in development, they may progressively exert a stronger influence on young adults' sense of satisfaction with their lives. We speculate that the more recent experience of these assets may be more salient in memory and more relevant to one's evolving identity, making them particularly influential when young people evaluate their lives at ages 19 and 21 years. Emerging adulthood is also characterized by increasing autonomy and important life decisions, including transitioning to postsecondary education, choosing a career, entering the workforce, and forming stable romantic relationships (Arnett, 2007). Internal or external assets available during the transition from adolescence to emerging adulthood may help consolidate adaptive coping strategies that support a successful entry into adulthood. These findings align with results from the Australian Temperament Project showing that strong family and peer relationships, as well as better emotional regulation between ages 15 and 18 years, are associated with positive development outcomes in young adulthood among individuals born in 1983 from a representative sample of Victoria, Australia (O'connor et al., 2011). Taken together, these results underscore the importance of continuing efforts to strengthen both internal and external assets as children and adolescents grow up, particularly given that life satisfaction tends to decline from early to late adolescence (Daly, 2022).

A similar pattern was observed for the association between internal assets and depression symptoms: internal assets measured closer in time to participants' reports on depression symptoms (ages 20–22 years) were more strongly associated with these symptoms, consistent with the recency hypothesis, although effect sizes were small. The capacity of internal assets to reduce the risk of depression symptoms appeared to increase with age. Examining individual internal asset categories, social competencies at ages 15 and 17 years were especially predictive of depressive symptoms. This finding suggests that promoting the development of social skills in later adolescence could be especially beneficial. Social skills facilitate supportive relationships, which may in turn prevent depressive symptoms by reducing social isolation and loneliness (Moeller & Seehuus, 2019; Scardera et al., 2020). They may also help adolescents cope better with stress and transitions during this period of major life changes by promoting effective communication, conflict resolution, and help‐seeking behaviors (Huttunen et al., 2025).

### Which assets matter most? Category‐level patterns

When examining individual asset categories separately, we found that only a few categories were uniquely and significantly associated with mental health outcomes in young adults. Some of these findings closely mirrored those observed for the broader asset dimensions (Internal and External), suggesting that certain categories may contribute disproportionally to the observed associations at the dimension level. For example, the recency hypothesis was selected for the association between internal assets and life satisfaction and depression symptoms—a pattern that was also observed for the Social Competencies category, which showed the strongest and most consistent associations across developmental periods relative to other internal assets (e.g., Commitment to Learning, Responsibility, Positive Identity). In contrast, some associations identified at the broader dimension level were not reflected in any single category. For example, the sensitive period hypothesis selected at age 12 years for the association between external assets and depression symptoms was not observed for any individual external asset categories. This pattern suggests that multiple external assets may collectively contribute to these associations. If replicated, such a finding would support prevention efforts targeting different types of external assets simultaneously across multiple contexts, including home, school, and community. However, these category‐level findings should be interpreted cautiously, as the broader asset dimensions were not modeled as multifactorial latent structures.

Furthermore, other associations revealed meaningful differences across the category‐ and dimension‐ level findings, suggesting that some asset categories may more robustly contribute to mental health outcomes. For example, Positive Identity measured at ages 12 and 13 years—an internal asset category primarily reflecting self‐esteem and a sense of purpose—was consistently associated with all four mental health outcomes. This finding highlights the potential importance of maintaining a positive self‐representation during the transition to secondary school. It is also consistent with findings of a German longitudinal study showing that youths entering adolescence (i.e., age 12 years) with higher self‐esteem and thereafter reporting increasing self‐esteem from ages 12 to 16 years, had significantly fewer depression symptoms at ages 16 and 35 years (Steiger et al., 2014). Together, these findings suggest that interventions aimed at fostering positive identity, such as programs designed to strengthen self‐esteem, promote goal setting, and support adolescents in developing a sense of purpose, may warrant further investigation during early adolescence and the transition to high school.

### Implications for prevention and intervention

While the findings of the present study should be considered preliminary, they point to potentially promising directions for future prevention research. External assets, such as support, safety, and clear boundaries, as well as positive identity (an internal asset), showed stronger associations with anxiety and depression symptoms when measured at the time of the transition to secondary school. However, given the observational design, small effect sizes, age‐related differences in measurement, and data‐adaptive analytic approach, these findings should not be interpreted as confirmatory. Future research should further examine the potential role of developmental timing in supporting or reinforcing internal assets, especially social competencies, during adolescence, as these assets appeared most closely linked to greater life satisfaction and lower depressive symptoms. Findings observed at the category level also suggest the potential importance of targeting multiple contexts (i.e., home, school, and community) and assets simultaneously. Taken together, and interpreted in light of these methodological limitations, the present findings provide preliminary evidence that relevance of developmental assets for later functioning may vary across development, potentially reflecting evolving needs, challenges, and resources from childhood through adolescence (Blakemore & Mills, 2014; Lupien et al., 2009).

### Strengths and limitations

The study offers a nuanced understanding of how promotive factors across development contribute to long‐term mental health, drawing on four key methodological strengths. First, it leverages a longitudinal design with nine repeated measures of assets collected annually or biennially over a 12‐year period (ages 5 to 17 years), allowing for detailed mapping of developmental timing across meaningful developmental stages. Second, it employs a comprehensive and multidimensional conceptualization of assets, incorporating both broad cumulative dimensions (internal and external assets) and more granular asset categories to clarify their distinct contributions. Third, the study integrates reports from multiple informants (mothers, teachers, interviewers, and the youth themselves), thereby enhancing measurement reliability and reducing single‐informant bias. Finally, it examines both positive and negative indicators of mental health, moving beyond a deficit‐based model to capture a more holistic understanding of functioning, aligned with the World Health Organization's definition of mental health (World Health Organization, 2022) and the dual framework of mental health (Keyes, 2005).

Several limitations should be considered when interpreting these findings. First, the number and content of items used to assess developmental assets varied across time points, and for some assets, were unavailable at certain ages (e.g., Responsibility at age 5). The asset indicators may have also varied over time because individual or external assets may be expressed differently across development and require to be measured with age‐appropriate indicators, consistent with the principle of heterotypic continuity. Notably, the means and distributions of the constructs remained similar after randomly selecting subsets of items (replicated 1000 times), indicating that the scores were minimally affected by the specific items selected (Thibault et al., in press). However, comparable distributions do not necessarily imply full conceptual consistency across age. Although conceptual continuity was promoted through the use of the same definitions, with items screened by the same research assistants, and subsequently selected and dichotomized by the same experts across the entire study period, these age‐specific differences may have limited the robustness of timing‐related findings. The contribution of different informants (e.g., teachers, mothers, youth), who may also be subject to distinct sources of bias, may have also influenced these findings. For example, mothers may have limited knowledge of their child's behavior or relationships at school, while teachers may have less insight into the child's behavior or resources outside the more structured classroom environment. This may have resulted in varying perspectives shaping the asset scores over time. Second, not all asset categories were equally well represented across development. Responsibility was assessed at eight out of the nine time points, Positive Identity at six, and Safety at four. However, all assets were measured at least four times over a span of at least 5 years (e.g., 12 to 17 years), allowing for comparison across different ages and developmental periods, except for Safety, which was measured only during adolescence. Third, although the QLSCD was representative of the Quebec population at its inception in the mid‐1990s, the sample consisted primarily of White, French‐speaking participants. Because the study began before major sociodemographic changes in Quebec, including higher maternal education, fewer children in low‐income households, overall improvements in socioeconomic conditions, and a greater proportion of immigrants (Orri et al., 2021), the findings may not generalize to more diverse or more recently born cohorts. Moreover, longitudinal attrition may have introduced additional bias. For example, participants who remained were more often females and, on average, from higher socioeconomic backgrounds. Although analyses adjusted for these differences, residual bias related to selective attrition may remain. Fourth, the multi‐factorial structure of the scales, incorporating all asset categories within a single model, was not tested in this study. This limits our ability to evaluate the joint structure of the asset dimensions and their shared variance, and to draw conclusions about the validity of the dimensional framework as a whole. This decision followed a theory‐driven approach, relying on the existing measurement framework to conceptualize developmental assets as partially overlapping and co‐occurring influences. At the same time, the framework aims to capture a wide range of influences that may not strongly overlap within individuals, which would be expected to result in lower shared variance across assets and categories at the category‐ and dimension‐levels, respectively. Accordingly, we prioritized a cumulative approach rather than deriving more unified and highly cohesive asset indicators.

## CONCLUSION

By repeatedly assessing developmental assets from ages 5 to 17 years, this study identifies windows when prevention and intervention may be most effective for promoting youth mental health. The findings emphasize that timing matters for both risk reduction and the promotion of well‐being. External assets present during the transition from primary to secondary school emerged as particularly important for reducing anxiety and depressive symptoms. Interventions that cultivate supportive relationships, ensure safety at school and in the community, encourage positive parenting practices, and promote constructive use of time through extracurricular activities, for example, may therefore be most impactful if implemented early in adolescence. In contrast, assets nurtured later in adolescence were more strongly linked to life satisfaction and happiness, highlighting the value of sustained investment in a wide range of internal and external assets, such as social competencies, positive identity, supportive relationships, and safety, as young people approach adulthood. Across all developmental periods, the consistent importance of social competencies and external support from families, schools, and neighborhoods underscores the need for effective intervention strategies that extend beyond the individual to engage families and community systems. Strengthening these relational and environmental resources at key developmental stages may yield lasting benefits for mental health across the transition to adulthood.

## AUTHOR CONTRIBUTIONS

**Geneviève Morneau‐Vaillancourt:** Conceptualization; investigation; writing – original draft; methodology; validation; visualization; writing – review and editing; formal analysis; data curation. **Marie‐Claude Geoffroy:** Resources; project administration; writing – review and editing; funding acquisition; investigation; conceptualization. **Maude Comtois‐Cabana:** Conceptualization; investigation; writing – review and editing. **Alexe Bilodeau‐Houle:** Data curation; writing – review and editing; conceptualization; investigation. **Michel Boivin:** Conceptualization; investigation; funding acquisition; writing – review and editing; project administration; resources. **Sylvana Côté:** Conceptualization; investigation; funding acquisition; writing – review and editing; project administration; resources. **Isabelle Ouellet‐Morin:** Conceptualization; investigation; funding acquisition; methodology; writing – review and editing; project administration; supervision; resources. **Mélodie Thibault:** Writing – review and editing; data curation; conceptualization; investigation. **Alexandra Matte‐Landry:** Conceptualization; investigation; funding acquisition; writing – review and editing.

## FUNDING INFORMATION

The QLSCD was supported by funding from the Ministère de la Santé et des services sociaux, Ministère de la Famille, Ministère de l'Éducation et de l'Enseignement supérieur, Ministère de l'Emploi et de la Solidarité sociale, Fondation Lucie and André Chagnon, Institut de recherche Robert‐Sauvé, Centre de recherche du CHU Sainte‐Justine, Institut de la statistique du Québec. Additional funding was received by the Fonds de Recherche du Québec—Santé (FRQS), the Fonds de Recherche du Québec—Société et Culture (FRQSC), the Social Sciences and Humanities Research Council of Canada (SSHRC), and the Canadian Institutes of Health Research (CIHR). All of these funding sources did not contribute to the study design, analysis of data, interpretation of results, or writing of the article.

## CONFLICT OF INTEREST STATEMENT

All authors declare no conflict of interest.

## ETHICS STATEMENT

Ethics committees of the *Institut de la Statistique du Québec* approved each phase of the study. Details about the QLSCD are provided in the cohort profile (Orri et al., 2021) and online: https://jesuisjeserai.stat.gouv.qc.ca. The secondary data analysis for this study was approved by the *Comité d'éthique de la recherche – Société et culture (CERSC)* of the Université de Montréal.

## PARTICIPANT CONSENT STATEMENT

Participants provided written informed consent at each data collection (parents and children's assent from age 10 years onward).

## Supporting information


**Table S1.** Number of items answered by each informant for each asset dimension and category.
**Table S2.** Pearson correlations between outcome variables.
**Table S3.** Multiple regression models predicting mental health outcomes from the internal and external dimension scales in the QLSCD.
**Table S4.** Multiple regression models predicting mental health outcomes from each asset categories.
**Table S5.** Multiple regression models predicting mental health outcomes from asset dimension scales by developmental periods.
**Table S6.** Multiple regression models predicting mental health outcomes from asset categories by developmental periods.
**Table S7.** SLCMA‐selected life‐course hypothesis for internal and external dimension scales by developmental periods.
**Table S8.** SLCMA‐selected life‐course hypothesis for each internal and external asset category by developmental periods.
**Figure S1.** Multiple regression models predicting mental health outcomes from the internal and external dimension scales in the QLSCD^a^ by developmental periods.
**Figure S2.** Multiple regression models predicting mental health outcomes from each asset categories in the QLSCD^a^ by developmental periods.

## Data Availability

The data that support the findings of this study are available on request from the corresponding author. The data are not publicly available due to privacy or ethical restrictions.
