# Supplementary material for: It's not just what you have, but when: The role and timing of developmental assets in the mental health of young adults
Source: J Res Adolesc. 2026 Jul 1;36(3):e70217. doi: 10.1111/jora.70217 (PMC13321777; doi:10.1111/jora.70217)
Supplement: Supplementary file 1 — Table S1. Number of items answered by each informant for each asset dimension and category. Table S2. Pearson correlations between outcome variables. Table S3. Multiple regression models predicting mental health outcomes from the internal and external dimension scales in the QLSCD. Table S4. Multiple regression models predicting mental health outcomes from each asset categories. Table S5. Multiple regression models predicting mental health outcomes from asset dimension scales by developmental periods. Table S6. Multiple regression models predicting mental health outcomes from asset categories by developmental periods. Table S7. SLCMA‐selected life‐course hypothesis for internal and external dimension scales by developmental periods. Table S8. SLCMA‐selected life‐course hypothesis for each internal and external asset category by developmental periods. Figure S1. Multiple regression models predicting mental health outcomes from the internal and external dimension scales in the QLSCDa by developmental periods. Figure S2. Multiple regression models predicting mental health outcomes from each asset categories in the QLSCDa by developmental periods. [file JORA-36-0-s001.docx]

**Supplementary Materials**

**It’s Not Just What You Have, But When: The Role and Timing of Developmental Assets in the Mental Health of Young Adults**

[Psychometric Properties of Asset Measures from Ages 5 to 10 Years 3](#_Toc231373394)

[Sensitivity Analyses for Happiness 4](#_Toc231373395)

[Multiple Imputation 6](#_Toc231373396)

[Table S1. Number of Items Answered by Each Informant for Each Asset Dimension and Category. 8](#_Toc231373397)

[Table S2. Pearson Correlations Between Outcome Variables 12](#_Toc231373398)

[Table S3. Multiple Regression Models Predicting Mental Health Outcomes from the Internal and External Dimension Scales in the QLSCD^a^ 14](#_Toc231373399)

[Table S4. Multiple Regression Models Predicting Mental Health Outcomes from Each Asset Categories 17](#_Toc231373400)

[Table S5. Multiple Regression Models Predicting Mental Health Outcomes from Asset Dimension Scales by Developmental Periods 22](#_Toc231373401)

[Table S6. Multiple Regression Models Predicting Mental Health Outcomes from Asset Categories by Developmental Periods 24](#_Toc231373402)

[Table S7. SLCMA-Selected Life-Course Hypothesis for Internal and External Dimension Scales by Developmental Periods 26](#_Toc231373403)

[Table S8. SLCMA-Selected Life-Course Hypothesis for Each Internal and External Asset Category by Developmental Periods 28](#_Toc231373404)

[Figure S1. Multiple Regression Models Predicting Mental Health Outcomes from the Internal and External Dimension Scales in the QLSCD^a^ by Developmental Periods 30](#_Toc231373405)

[Figure S2. Multiple Regression Models Predicting Mental Health Outcomes from each Asset Categories in the QLSCD^a^ by Developmental Periods 32](#_Toc231373406)

[References 34](#_Toc231373407)

**Supplementary Materials**

The supplementary materials provide additional information on the asset measures, sensitivity analyses for happiness, and the multiple imputation procedure. They also include supplementary tables and figures presenting descriptive statistics, correlations among outcomes, and results from sensitivity analyses.

# Psychometric Properties of Asset Measures from Ages 5 to 10 Years

Internal consistency was evaluated using mean inter-item correlations (MIIC) as most assets were derived using less than 10 items, with optimal MIIC suggested to be between .20 and .40 (Briggs & Cheek, 1986). Most assets showed a MIIC > .15, and those who showed smaller correlations included items evaluated by different informants across distinct contexts (e.g., mothers evaluating the child at home versus teachers evaluating the child at school). To assess the unidimensional nature of the asset categories including four or more individual assets, we conducted confirmatory factor analyses using the lavaan package in R, as described in Thibault et al. (in press). Fit was evaluated using the Comparative Fit Index (CFI; Bentler, 1990) and the Tucker-Lewis Index (TLI; Tucker & Lewis, 1973), with values higher than .90 indicating good fit. We also used the Root Mean Square Error of Approximation (RMSEA; MacCallum et al., 1996) and the Standardized Root Mean Residual (SRMR; Hu & Bentler, 1999), with values less than .08 indicating an acceptable fit. Between ages 5 and 10 years, six asset categories included at least four individual assets (Support at 6, 8, and 10 years old; Social competencies at 8 and 10 years old; Boundaries and Expectations at 10 years old). Overall, our results mirror those obtained in Thibault et al. (in press). Only 1/6 categories, Social Competencies at 8 years old, showed less-than-optimal fit: CFI = .91; TLI = .73; RMSEA = .14; SRMR = .05. However, after adding a covariance parameter between two asset residuals (Planning and Interpersonal Competencies), the model fit was optimal: CFI = .99; TLI = .99; RMSEA = .03; SRMR = .01. We added this covariance parameter based on modification indices and because the assets Planning and Interpersonal Competencies were primarily evaluated by teachers, whereas other assets in the Social Competencies category, such as Resistance Skills, were partly evaluated by mothers. Given that different raters contributed to the evaluation of items within the overall asset category, it was justified to allow a covariance between the residuals of the two assets rated by the same rater (teacher). The other five categories showed adequate fit (CFI and TLI > .90; RMSEA and SMRM < .08), supporting the aggregation of these assets into cumulative unidimensional categories captured across informants and contexts. Finally, we examined the stability of asset categories over time and found that they were moderately stable from 5 to 10 years old, as indicated by Pearson correlations larger than .29 on average.

# Sensitivity Analyses for Happiness

Results of the multiple regression analyses predicting happiness are presented in Table S3, S4, S5, S6, and Figure S1 and S2. With respect to the main SLCMA analyses conducted with internal and external asset dimensions, results are the same as those for life satisfaction in Table 2 (same coefficients). We present the results of the SLCMA analyses conducted with individual asset categories in the table below. Results of the SLCMA analyses by developmental periods are presented in Table S7 and S8.

| SLCMA-Selected Life-Course Hypothesis for Internal and External Asset Categories Predicting Happiness | | | |
| --- | --- | --- | --- |
|  | Happiness 19-21 years | | |
|  | Step 1 | | Step 2 |
|  | Model | R^2^ | Estimate (95% CI) |
| Commitment to Learning | **~~-~~** | **~~-~~** | **~~-~~** |
| Responsibility | - | - | - |
| Social Competencies | Recency | .013 | **.001 (.001, .002)** |
|  | 17 years | .050 | **.057 (.014, .092)** |
| Positive Identity | 13 years | .016 | **.060 (.039, .080)** |
|  | 12 years | .056 | **.045 (.024, .066)** |
| Support | Recency | .022 | **.002 (.001, .002)** |
| Safety | - | - | - |
|  |  |  |  |
| Boundaries and Expectations | ~~-~~ | ~~-~~ | **~~-~~** |
| Constructive Use of Time | Recency | .008 | **.001 (.000, .002)** |
| *Notes.* Dashes indicate SLCMA models that were not tested because multiple regression results showed no significant association between the asset category and the outcome. Step 1 corresponds to the Least Angle Regression (LARS) models. Step 2 corresponds to the post-selection inference. Estimates in bold were significant according to a FDR-adjusted *p-*value < .05. R^2^ represents the proportion of variance in mental health outcomes explained by the developmental assets, according to the selected hypothesis and after adjusting for covariates (sex, socioeconomic status, parental origin, family type, externalizing and internalizing problems in early childhood). For the recency hypothesis, the estimate from step 2 represents the change in the mental health outcome z-score for a one-point increase in exposure, weighted by the temporal proximity of exposure to the outcome. For the time-specific, sensitive period hypothesis, the estimate reflects the difference in the outcome z-score between individuals exposed vs. unexposed at this specific age.  Abbreviations: SLCMA = Structured Life-course Modeling Approach; CI = confidence intervals.  Data were compiled from the final master file of the Quebec Longitudinal Study of Child Development (1998–2018), ©Gouvernement du Québec, Institut de la statistique du Québec. | | | |

# Multiple Imputation

There were 1344 participants with complete outcome data in our analyses, including some with missing data for developmental assets or confounding variables (see Table 1). The number of participants available varied across variables and time points due to attrition and to some asset items having to be eliminated as they did not discriminate well enough between youths who were exposed to the asset and those who were not (i.e., most participants answered "yes").

We performed multiple imputation to reduce bias due to missing data and maximize the use of available data. We used multiple chain equations as implemented in the mice package version 3.16.0 in R version 4.2.3 (Azur et al., 2011; R Core Team, 2023; van Buuren et al., 1999, van Buuren and Groothuis-Oudshoorn, 2011). We performed multiple imputation four times as we used different asset conceptualizations, creating four sets of 20 imputed datasets (with 25 iterations each) that were used in the main analyses. The four separate imputation procedures were conducted for 1) asset dimension scales evaluated at each time point (Internal, External), 2) asset categories evaluated at each time point (Commitment to Learning, Responsibility, Social Competencies, Positive Identity, Support, Safety, Boundaries and Expectations, and Constructive Use of Time), 3) asset dimension scales combined into developmental periods, and 4) asset categories combined into developmental periods. For all four imputation procedures, we imputed the following confounding variables: Canadian origin of the mother and father, family type, socioeconomic status, children's hyperactivity, aggression, and emotional problems (see Methods for a description of the covariates). We did not impute child's sex as a covariate because no data was missing for this variable. All variables were imputed using predictive mean matching, except for the binary covariates (origin of the mother and father, family type), which were imputed using logistic regression. To assess the quality of imputation, we compared imputed data to observed data by examining density plots, means, standard deviations, proportions of cases, and correlations between variables. Estimates were similar across imputed and observed variables for all four imputed datasets.

| Table S1. Number of Items Answered by Each Informant for Each Asset Dimension and Category. | | | | | | | | | | | |
| --- | --- | --- | --- | --- | --- | --- | --- | --- | --- | --- | --- |
|  |  | **5 years** | **6 years** | **7 years** | **8 years** | **10 years** | **12 years** | **13 years** | **15 years** | **17 years** |  |
|  | ***Informant*** | ***Number of items*** | | | | | | | | | ***Total*** |
| **Internal dimension** | Child | 0 | 3 | 11 | 12 | 20 | 29 | 35 | 36 | 34 | 180 |
|  | Parent | 4 | 16 | 0 | 12 | 1 | 0 | 1 | 0 | 0 | 34 |
|  | Teacher | 7 | 25 | 28 | 26 | 26 | 18 | 19 | 0 | 0 | 149 |
|  | Interviewer | 0 | 1 | 5 | 1 | 0 | 1 | 1 | 0 | 0 | 9 |
|  | **Total** | **11** | **45** | **44** | **51** | **47** | **48** | **56** | **36** | **34** | **372** |
| Commitment to Learning | Child | 0 | 3 | 11 | 12 | 10 | 16 | 16 | 16 | 16 | 100 |
|  | Parent | 4 | 5 | 0 | 2 | 1 | 0 | 1 | 0 | 0 | 13 |
|  | Teacher | 0 | 6 | 5 | 5 | 5 | 7 | 12 | 0 | 0 | 40 |
|  | Interviewer | 0 | 0 | 4 | 0 | 0 | 0 | 0 | 0 | 0 | 4 |
|  | **Total** | **4** | **14** | **20** | **19** | **16** | **23** | **29** | **16** | **16** | **157** |
| Responsibility | Child | 0 | 0 | 0 | 0 | 0 | 0 | 0 | 2 | 2 | 4 |
|  | Parent | 0 | 0 | 0 | 0 | 0 | 0 | 0 | 0 | 0 | 0 |
|  | Teacher | 0 | 1 | 1 | 1 | 1 | 1 | 1 | 0 | 0 | 6 |
|  | Interviewer | 0 | 0 | 0 | 0 | 0 | 0 | 0 | 0 | 0 | 0 |
|  | **Total** | 0 | **1** | **1** | **1** | **1** | **1** | **1** | **2** | **2** | **10** |
| Social Competencies | Child | 0 | 0 | 0 | 0 | 10 | 11 | 10 | 18 | 16 | 65 |
|  | Parent | 0 | 11 | 0 | 10 | 0 | 0 | 0 | 0 | 0 | 21 |
|  | Teacher | 7 | 17 | 21 | 19 | 19 | 10 | 4 | 0 | 0 | 97 |
|  | Interviewer | 0 | 0 | 0 | 0 | 0 | 0 | 0 | 0 | 0 | 0 |
|  | **Total** | **7** | **28** | **21** | **29** | **29** | **21** | **14** | **18** | **16** | **183** |
| Positive Identity | Child | 0 | 0 | 0 | 0 | 0 | 2 | 9 | 0 | 0 | 11 |
|  | Parent | 0 | 0 | 0 | 0 | 0 | 0 | 0 | 0 | 0 | 0 |
|  | Teacher | 0 | 1 | 1 | 1 | 1 | 0 | 2 | 0 | 0 | 6 |
|  | Interviewer | 0 | 1 | 1 | 1 | 0 | 1 | 1 | 0 | 0 | 5 |
|  | **Total** | **0** | **2** | **2** | **2** | **1** | **3** | **12** | **0** | **0** | **22** |
| **External dimension** | Child | 0 | 5 | 6 | 6 | 13 | 18 | 24 | 22 | 21 | 115 |
|  | Parent | 28 | 38 | 24 | 32 | 28 | 21 | 32 | 34 | 35 | 272 |
|  | Teacher | 0 | 11 | 10 | 9 | 6 | 6 | 14 | 0 | 0 | 56 |
|  | Interviewer | 0 | 0 | 0 | 0 | 0 | 0 | 0 | 0 | 0 | 0 |
|  | **Total** | **28** | **54** | **40** | **47** | **47** | **45** | **70** | **56** | **56** | **443** |
| Support | Child | 0 | 4 | 4 | 4 | 7 | 13 | 17 | 15 | 14 | 78 |
|  | Parent | 11 | 22 | 14 | 13 | 13 | 13 | 23 | 23 | 24 | 156 |
|  | Teacher | 0 | 5 | 4 | 4 | 3 | 3 | 4 | 0 | 0 | 23 |
|  | Interviewer | 0 | 0 | 0 | 0 | 0 | 0 | 0 | 0 | 0 | 0 |
|  | **Total** | **11** | **31** | **22** | **21** | **23** | **29** | **44** | **38** | **38** | **257** |
| Safety | Child | 0 | 0 | 0 | 0 | 0 | 1 | 1 | 1 | 1 | 4 |
|  | Parent | 0 | 0 | 0 | 0 | 0 | 0 | 0 | 0 | 0 | 0 |
|  | Teacher | 0 | 0 | 0 | 0 | 0 | 0 | 2 | 0 | 0 | 2 |
|  | Interviewer | 0 | 0 | 0 | 0 | 0 | 0 | 0 | 0 | 0 | 0 |
|  | **Total** | **0** | **0** | **0** | **0** | **0** | **1** | **3** | **1** | **1** | **6** |
| Boundaries and Expectations | Child | 0 | 1 | 2 | 2 | 6 | 3 | 4 | 4 | 4 | 26 |
|  | Parent | 8 | 5 | 1 | 6 | 4 | 4 | 6 | 8 | 8 | 50 |
|  | Teacher | 0 | 6 | 6 | 5 | 3 | 3 | 8 | 0 | 0 | 31 |
|  | Interviewer | 0 | 0 | 9 | 0 | 0 | 0 | 0 | 0 | 0 | 9 |
|  | **Total** | **8** | **12** | **18** | **13** | **13** | **10** | **18** | **12** | **12** | **116** |
| Constructive Use of Time | Child | 0 | 0 | 0 | 0 | 0 | 1 | 2 | 2 | 2 | 7 |
|  | Parent | 9 | 11 | 9 | 13 | 11 | 4 | 3 | 3 | 3 | 66 |
|  | Teacher | 0 | 0 | 0 | 0 | 0 | 0 | 0 | 0 | 0 | 0 |
|  | Interviewer | 0 | 0 | 0 | 0 | 0 | 0 | 0 | 0 | 0 | 0 |
|  | **Total** | **9** | **11** | **9** | **13** | **11** | **5** | **5** | **5** | **5** | **73** |
| *Note*. Data were compiled from the final master file of the Québec Longitudinal Study of Child Development (2010–2015), ©Gouvernement du Québec, Institut de la statistique du Québec. | | | | | | | | | | | |

| Table S2. Pearson Correlations Between Outcome Variables | | | | | | | | |
| --- | --- | --- | --- | --- | --- | --- | --- | --- |
| Mental Health Outcomes Measured at Each Time Point | | | | | | | | |
|  | 1. | 2. | 3. | 4. | 5. | 6. | 8. | 9. |
| 1. Life satisfaction 19 years | 1 |  |  |  |  |  |  |  |
| 2. Life satisfaction 21 years | .52  (.47, .56) | 1 |  |  |  |  |  |  |
| 3. Happiness 19 years | .81  (.79, .83) | .48  (.44, .53) | 1 |  |  |  |  |  |
| 4. Happiness 21 years | .48  (.43, .52) | .79  (.77, .81) | .49  (.44, .53) | 1 |  |  |  |  |
| 5. Anxiety symptoms 20 years | -.36  (-.41, -.31) | -.38  (-.42, -.32) | -.34  (-.39, -.28) | -.38  (-.43, -.33) | 1 |  |  |  |
| 6. Anxiety symptoms 22 years | -.26  (-.31, -.20) | -.33  (-.38, -.28) | -.26  (-.31, -.20) | -.34  (-.39, -.29) | .49  (.44, .53) | 1 |  |  |
| 8. Depression symptoms 20 years | -.48  (-.52, -.43) | -.50  (-.54, .45) | -.48  (-.52, -.43) | -.52  (-.56, -.48) | .63  (.59, .66) | .38  (.33, .43) | 1 |  |
| 9. Depression symptoms 22 years | -.37  (-.42, -.32) | -.44  (-.48, -.39) | -.36  (-.41, -.31) | -.47  (-.51, -.42) | .40  (.35, .45) | .70  (.67, .73) | .51  (.46, .55) | 1 |
| Mental Health Outcomes Averaged Across Time Points | | | | | | | | |
|  | Life satisfaction 19-21 years | | Happiness 19-21 years | | Anxiety symptoms 20-22 years | | Depression symptoms 20-22 years | |
| 1. Life satisfaction 19-21 years | 1 | |  | |  | |  | |
| 2. Happiness 19-21 years | .85  (.83, .91) | | 1 | |  | |  | |
| 3. Anxiety symptoms 20-22 years | -.39  (-.44, -.34) | | -.39  (-.44, -.34) | | 1 | |  | |
| 4. Depression symptoms 20-22 years | -.55  (-.58, -.51) | | -.56  (-.60, -.52) | | .70  (.68, .73) | | 1 | |
| *Note*. Data were compiled from the final master file of the Québec Longitudinal Study of Child Development (2010–2015), ©Gouvernement du Québec, Institut de la statistique du Québec. | | | | | | | | |

| Table S3. Multiple Regression Models Predicting Mental Health Outcomes from the Internal and External Dimension Scales in the QLSCD^a^ | | | | | | | | |
| --- | --- | --- | --- | --- | --- | --- | --- | --- |
|  | **Life satisfaction 19-21 years** | | **Happiness 19-21 years** | | **Anxiety symptoms 20-22 years** | | **Depressive symptoms 20-22 years** | |
|  | **B (95% CI)** | **p-value** | **B (95% CI)** | **p-value** | **B (95% CI)** | **p-value** | **B (95% CI)** | **p-value** |
| **Model 1 – 5 years** |  |  |  |  |  |  |  |  |
| Internal assets | .08 (.02, .14) | .01* | .04 (-.02, .10) | .20 | -.01 (-.07, .05) | .80 | -.03 (-.09, .03) | .27 |
| External assets | .01 (-.05, .06) | .81 | .03 (-.03, 08) | .34 | .01 (-.05, .06) | .84 | -.03 (-.08, .03) | .31 |
| **Model 2 – 6 years** |  |  |  |  |  |  |  |  |
| Internal assets | .12 (.04, .19) | <.01* | .10 (.02, .19) | .02* | -.14 (-.21, -.07) | <.01* | -.19 (-.27, -.11) | <.01* |
| External assets | .06 (-.01, .12) | .08 | .07 (.01, .13) | .02* | .03 (-.03, .09) | .26 | .01 (-.05, .07) | .78 |
| **Model 3 – 7 years** |  |  |  |  |  |  |  |  |
| Internal assets | .12 (.05, .19) | <01* | .08 (.01, .14) | .02* | -.07 (-.13, -.01) | .02* | -.09 (-.16, -.03) | <.01* |
| External assets | .04 (-.02, .10) | .22 | .04 (-.02, .10) | .19 | .01 (-.05, .07) | .80 | -.05 (-.11, .01) | .12 |
| **Model 4 – 8 years** |  |  |  |  |  |  |  |  |
| Internal assets | .13 (.06, .20) | <.01* | .09 (.02, .15) | .01* | -.08 (-.14, -.01) | .02* | -.12 (-.19, -.06) | <.01* |
| External assets | .09 (.02, .16) | .01* | .13 (.06, .19) | <.01* | -.04 (-.10, .03) | .26 | -.09 (-.15, -.02) | <.01* |
| **Model 5 – 10 years** |  |  |  |  |  |  |  |  |
| Internal assets | .16 (.07, .24) | <.01* | .08 (.00, .17) | .05 | -.06 (-.13, .01) | .08 | -.11 (-.18, -.04) | <.01* |
| External assets | .08 (.02, .14) | <.01* | .11 ( .05, .17) | <.01* | -.06 (-.12, .00) | .06 | -.12 (-.18, -.06) | <.01* |
| **Model 6 – 12 years** |  |  |  |  |  |  |  |  |
| Internal assets | .18 (.12, .25) | <.01* | .13 (.06, .21) | <.01* | -.07 (-.15, .01) | .07 | -.13 (-.20, -.06) | <.01* |
| External assets | .09 (.03, .16) | <.01* | .12 (.05, .18) | <.01* | -.09 (-.16, -.02) | <.01* | -.14 (-.21, -.07) | <.01* |
| **Model 7 – 13 years** |  |  |  |  |  |  |  |  |
| Internal assets | .17 (.10, .24) | <.01* | .11 (.04, .18) | <.01* | -.07 (-.13, -.01) | .03* | -.14 (-.21, -.08) | <.01* |
| External assets | .08 (.01, .15) | .02* | .13 (.06, .19) | <.01* | -.04 (-.10, .02) | .20 | -.09 (-.15, -.02) | .01* |
| **Model 8 – 15 years** |  |  |  |  |  |  |  |  |
| Internal assets | .11 (.05, .18) | <.01* | .09 (.03, .15) | <.01* | -.04 (-.10, .02) | .18 | -.11 (-.17, -.05) | <.01* |
| External assets | .12 (.05, .18) | <.01* | .13 (.07, .20) | <.01* | -.08 (-.14, -.02) | .01* | -.09 (-.15, -.03) | <.01* |
| **Model 9 – 17 years** |  |  |  |  |  |  |  |  |
| Internal assets | .14 (.07, .20) | <.01* | .14 (.08, .20) | <.01* | -.08 (-.14, -.02) | .01* | -.15 (-.21, -.09) | <.01* |
| External assets | .14 (.08, .21) | <.01* | .16 (.10, .23) | <.01* | -.05 (-.12, .01) | .10 | -.11 (-.17, -.04) | <.01* |
| *Notes*. Estimates are z-standardized. Internal and External dimension scales were entered together in all models. Models were adjusted for covariates: sex, socioeconomic status, parent origin, family type, externalizing and internalizing problems in early childhood.  Abbreviations: QLSCD = Quebec Longitudinal Study of Child Development.  ^a^Data were compiled from the final master file of the Quebec Longitudinal Study of Child Development (1998–2018), ©Gouvernement du Québec, Institut de la statistique du Québec.  * *p* < .05 after false-discovery rate correction. | | | | | | | | |

| Table S4. Multiple Regression Models Predicting Mental Health Outcomes from Each Asset Categories | | | | | | | | |
| --- | --- | --- | --- | --- | --- | --- | --- | --- |
|  | **Life satisfaction**  **19-21 years** | | **Happiness**  **19-21 years** | | **Anxiety symptoms**  **20-22 years** | | **Depressive symptoms**  **20-22 years** | |
|  | **B (95% CI)** | **p-value** | **B (95% CI)** | **p-value** | **B (95% CI)** | **p-value** | **B (95% CI)** | **p-value** |
| **Model 1 – 5 years** |  |  |  |  |  |  |  |  |
| *Internal assets* |  |  |  |  |  |  |  |  |
| Commitment to Learning | .02 (-.04, .08) | .57 | .01 (-.05, .07) | .72 | .08 (.02, .14) | .01* | .02 (-.04, .08) | .46 |
| Social Competencies | .09 (.03, .15) | <.01* | .05 (-.01, .11) | .11 | -.06 (-.12, -.00) | .05 | -.06 (-.12, .00) | .05 |
| *External assets* |  |  |  |  |  |  |  |  |
| Support | .03 (-.03, .09) | .35 | .03 (-.03, .09) | .34 | -.02 (-.08, .04) | .48 | -.05 (-.10, .01) | .12 |
| Boundaries and Expectations | -.03 (-.09, .02) | .25 | -.00 (-.06, .06) | 1.00 | .00 (-.05, .06) | .93 | .02 (-.03, .08) | .38 |
| Constructive Use of Time | .03 (-.03, .09) | .39 | .02 (-.04, .07) | .59 | .01 (-.05, .07) | .70 | -.03 (-.09, .02) | .25 |
| **Model 2 – 6 years** |  |  |  |  |  |  |  |  |
| *Internal assets* |  |  |  |  |  |  |  |  |
| Commitment to Learning | .02 (-.06, .09) | .68 | .01 (-.06, .08) | .76 | -.01 (-.09, .06) | .70 | -.05 (-.12, .03) | .23 |
| Responsibility | .00 (-.08, .08) | .97 | -.01 (-.10, .07) | .73 | -.00 (-.09, .09) | .97 | .02 (-.07, .11) | .62 |
| Social Competencies | .11 (.03, .19) | .01* | .11 (.01, .20) | .03 | -.14 (-.23, -.05) | <.01* | -.19 (-.28, -.10) | <.01* |
| *External assets* |  |  |  |  |  |  |  |  |
| Support | .07 (-.00, .14) | .05 | .08 (.01, .15) | .03 | .02 (-.04, .08) | .54 | -.02 (-.08, .04) | .53 |
| Boundaries and Expectations | .01 (-.07, .08) | .88 | .01 (-.06, .08) | .73 | .01 (-.06, .08) | .76 | .01 (-.07, .08) | .89 |
| Constructive Use of Time | -.00 (-.07, .06) | .89 | -.01 (-.07, .05) | .82 | .00 (-.06, .06) | .94 | .03 (-.03, .09) | .32 |
| **Model 3 – 7 years** |  |  |  |  |  |  |  |  |
| *Internal assets* |  |  |  |  |  |  |  |  |
| Commitment to Learning | .07 (-.00, .14) | .06 | .04 (-.03, .12) | .24 | -.03 (-.10, .04) | .40 | -.06 (-.13, .01) | .10 |
| Responsibility | .06 (-.01, .14) | .09 | .05 (-.02, .12) | .15 | -.03 (-.10, .04) | .44 | -.04 (-.12, .03) | .21 |
| Social Competencies | .04 (-.05, .13) | .36 | .03 (-.06, .12) | .46 | -.04 (-.13, .04) | .32 | -.04 (-.12, .05) | .40 |
| Positive Identity | -.03 (-.09, .03) | .38 | -.03 (-.10, .03) | .25 | -.00 (-.06, .06) | .99 | -.00 (-.07, .06) | .90 |
| *External assets* |  |  |  |  |  |  |  |  |
| Support | .04 (-.02, .11) | .18 | .05 (-.02, .11) | .16 | .01 (-.05, .07) | .78 | -.02 (-.08, .04) | .45 |
| Boundaries and Expectations | .00 (-.06, .07) | .90 | .01 (-.06, .07) | .83 | -.01 (-.07, .05) | .66 | -.03 (-.09, .03) | .35 |
| Constructive Use of Time | .02 (-.05, .08) | .61 | .02 (-.04, .08) | .55 | .01 (-.05, .07) | .79 | -.04 (-.10, .01) | .14 |
| **Model 4 – 8 years** |  |  |  |  |  |  |  |  |
| *Internal assets* |  |  |  |  |  |  |  |  |
| Commitment to Learning | .08 (.00, .16) | .05 | .08 (-.01, .16) | .07 | -.06 (-.12, .02) | .14 | -.08 (-.15, -.01) | .03 |
| Responsibility | .02 (-.06, .09) | .65 | .04 (-.03, .10) | .30 | -.01 (-.06, .07) | .92 | .00 (-.06, .07) | .89 |
| Social Competencies | .07 (-.02, .15) | .11 | .02 (-.06, .10) | .68 | -.04 (-.14, .02) | .13 | -.09 (-.17, -.01) | .03 |
| Positive Identity | .01 (-.06, .08) | .88 | -.01 (-.07, .06) | .87 | .02 (-.04, .09) | .45 | .01 (-.06, .08) | .78 |
| *External assets* |  |  |  |  |  |  |  |  |
| Support | .07 (.01, .14) | .03 | .09 (.02, .15) | .01* | -.01 (-.07, .06) | .87 | -.03 (-.09, .04) | .41 |
| Boundaries and Expectations | -.03 (-.09, .04) | .40 | -.01 (-.08, .06) | .74 | .02 (-.04, .09) | .39 | -.00 (-.07, .06) | .88 |
| Constructive Use of Time | .08 (.01, .15) | .02 | .09 (.03, .15) | <.01* | -.07 (-.04, .09) | .03 | -.11 (-.17, -.04) | <.01* |
| **Model 5 – 10 years** |  |  |  |  |  |  |  |  |
| *Internal assets* |  |  |  |  |  |  |  |  |
| Commitment to Learning | .06 (-.01, .13) | .11 | .02 (-.06, .09) | .63 | .00 (-.07, .07) | .91 | -.06 (-.13, .02) | .13 |
| Responsibility | .02 (-.06, .09) | .69 | .02 (-.06, .10) | .61 | .01 (-.07, .08) | .87 | .02 (-.05, .10) | .55 |
| Social Competencies | .12 (.01, .24) | .04 | .10 (.00, .20) | .05 | -.08 (-.18, .01) | .09 | -.13 (-.22, -.03) | .01* |
| Positive Identity | -.02 (-.09, .05) | .63 | -.03 (-.10, .04) | .44 | -.02 (-.09, .05) | .67 | .02 (-.05, .08) | .66 |
| *External assets* |  |  |  |  |  |  |  |  |
| Support | .04 (-.04, .11) | .33 | .03 (-.04, .10) | .43 | -.05 (-.11, .01) | .14 | -.04 (-.11, .02) | .22 |
| Boundaries and Expectations | -.00 (-.06, .06) | .96 | .01 (-.05, .08) | .68 | .04 (-.02, .11) | .16 | .01 (-.05, .08) | .84 |
| Constructive Use of Time | .08 (.02, .15) | .02 | .11 (.04, .18) | <.01* | -.05 (-.11, .01) | .08 | -.13 (-.18, -.07) | <.01* |
| **Model 6 – 12 years** |  |  |  |  |  |  |  |  |
| *Internal assets* |  |  |  |  |  |  |  |  |
| Commitment to Learning | -.00 (-.09, .08) | .93 | -.04 (-.13, .04) | .31 | .04 (-.04, .12) | .28 | .04 (-.03, .12) | .27 |
| Responsibility | -.02 (-.09, .05) | .49 | -.04 (-.12, .04) | .33 | .01 (-.07, .09) | .86 | -.01 (-.08, .07) | .87 |
| Social Competencies | .12 (.01, .22) | .03 | .11 (.00, .23) | .05 | -.03 (-.14, .06) | .45 | -.06 (-.15, .04) | .24 |
| Positive Identity | .14 (.07, .21) | <.01* | .13 (.06, .20) | <.01* | -.07 (-.13, -.01) | .02 | -.14 (-.20, -.07) | <.01* |
| *External assets* |  |  |  |  |  |  |  |  |
| Support | .11 (.04, .17) | <.01* | .12 ( .06, .19) | <.01* | -.10 (-.17, -.04) | <.01* | -.11 (-.17, -.05) | <.01* |
| Safety | .03 (-.03, .10) | .32 | .04 (-.03, .10) | .29 | -.08 (-.15, -.02) | .01* | -.12 (-.18, -.06) | <.01* |
| Boundaries and Expectations | -.02 (-.08, .04) | .56 | -.03 (-.09, .04) | .42 | .03 (-.02, .10) | .31 | -.01 (-.07, .05) | .72 |
| Constructive Use of Time | .02 (-.04, .09) | .52 | .04 (-.03, .10) | .27 | -.02 (-.09, .04) | .56 | -.04 (-.10, .02) | .22 |
| **Model 7 – 13 years** |  |  |  |  |  |  |  |  |
| *Internal assets* |  |  |  |  |  |  |  |  |
| Commitment to Learning | .02 (-.07, .11) | .70 | -.02 (-.12, .07) | .63 | .05 (-.04, .15) | .26 | .01 (-.08, .10) | .82 |
| Responsibility | -.05 (-.12, .03) | .24 | -.04 (-.12, .04) | .38 | -.02 (-.10, .06) | .68 | .04 (-.03, .12) | .23 |
| Social Competencies | .07 (-.00, .14) | .06 | .04 (-.03, .12) | .26 | -.03 (-.11, .05) | .43 | -.05 (-.12, .03) | .26 |
| Positive Identity | .18 (.11, .25) | <.01* | .17 (.10, .24) | <.01* | -.12 (-.19, -.05) | <.01* | -.23 (-.30, -.16) | <.01* |
| *External assets* |  |  |  |  |  |  |  |  |
| Support | .14 (.06, .21) | <.01* | .17 (.09, .25) | <.01* | -.06 (-.13, .01) | .09 | -.08 (-.15, -.00) | .04 |
| Safety | -.01 (-.07, .05) | .79 | -.01 ( -.07, .05) | .74 | -.04 (-.10, .03) | .25 | -.01 (-.07, .05) | .70 |
| Boundaries and Expectations | -.09 (-.16, -.02) | .01* | -.08 (-.15, -.01) | .02 | .08 (.02, .14) | .02 | .07 (.00, .13) | .04 |
| Constructive Use of Time | -.03 (-.09, .04) | .45 | -.01 (-.08, .06) | .84 | -.01 (-.08, .05) | .67 | -.02 (-.09, .05) | .50 |
| **Model 8 – 15 years** |  |  |  |  |  |  |  |  |
| *Internal assets* |  |  |  |  |  |  |  |  |
| Commitment to Learning | -.00 (-.07, .06) | .90 | -.02 (-.09, .05) | .53 | .11 (.05, .18) | <.01* | .07 (.01, .14) | .03 |
| Responsibility | -.02 (-.08, .04) | .45 | -.03 (-.09, .03) | .36 | .05 (-.00, .11) | .06 | .03 (-.03, .09) | .31 |
| Social Competencies | .11 (.05, .17) | <.01* | .10 (.04, .17) | <.01* | -.13 (-.19, -.07) | <.01* | -.16 (-.22, -.10) | <.01* |
| *External assets* |  |  |  |  |  |  |  |  |
| Support | .15 (.07, .22) | <.01* | .16 (.08, .24) | <.01* | -.07 (-.15, .01) | .08 | -.08 (-.16, -.01) | .03 |
| Safety | .07 (.01, .13) | .03 | .06 (.00, .12) | .05 | -.11 (-.17, -.05) | <.01* | -.11 (-.17, -.05) | <.01* |
| Boundaries and Expectations | -.04 (-.11, .04) | .35 | -.04 (-.12, .03) | .26 | -.00 (-.07, .07) | .94 | -.01 (-.08, .06) | .86 |
| Constructive Use of Time | .03 (-.05, .10) | .35 | .05 (-.02, .11) | .14 | .02 (-.05, .08) | .61 | -.02 (-.08, .04) | .48 |
| **Model 9 – 17 years** |  |  |  |  |  |  |  |  |
| *Internal assets* |  |  |  |  |  |  |  |  |
| Commitment to Learning | .03 (-.04, .10) | .39 | .02 (-.05, .09) | .51 | .07 (.00, .14) | .03 | .04 (-.03, .10) | .25 |
| Responsibility | -.05 (-.10, .01) | .10 | -.03 (-.08, .03) | .39 | .10 (.05, .17) | <.01* | .06 (-.00, .11) | .06 |
| Social Competencies | .13 (.07, .19) | <.01* | .14 (.08, .21) | <.01* | -.17 (-.23, -.11) | <.01* | -.22 (-.28, -.16) | <.01* |
| *External assets* |  |  |  |  |  |  |  |  |
| Support | .17 (.08, .27) | <.01* | .21 (.12, .29) | <.01* | -.09 (-.17, -.00) | .03 | -.13 (-.21, -.05) | <.01* |
| Safety | .08 (.02, .14) | <.01* | .05 (-.01, .12) | .09 | -.06 (-.12, .00) | .06 | -.08 (-.14, -.02) | .01* |
| Boundaries and Expectations | -.07 (-.15, .02) | .12 | -.08 (-.16, .00) | .06 | .04 (-.04, .10) | .33 | .03 (-.04, .11) | .38 |
| Constructive Use of Time | .02 (-.07, .11) | .72 | -.01 (-.09, .07) | .82 | .05 (-.02, .12) | .16 | .04 (-.04, .12) | .38 |
| *Notes*. Estimates are z-standardized. All asset categories were entered together in the same model. Models were adjusted for covariates (sex, socioeconomic status, parental origin, family type, externalizing and internalizing problems in early childhood).  Data were compiled from the final master file of the Quebec Longitudinal Study of Child Development (1998-2020), ©Gouvernement du Québec, Institut de la statistique du Québec.  * *p* < .05 after false-discovery rate correction. | | | | | | | | |

| Table S5. Multiple Regression Models Predicting Mental Health Outcomes from Asset Dimension Scales by Developmental Periods | | | | | | | | |
| --- | --- | --- | --- | --- | --- | --- | --- | --- |
|  | **Life satisfaction 19-21 years** | | **Happiness 19-21 years** | | **Anxiety symptoms 20-22 years** | | **Depressive symptoms 20-22 years** | |
|  | **B (95% CI)** | **p-value** | **B (95% CI)** | **p-value** | **B (95% CI)** | **p-value** | **B (95% CI)** | **p-value** |
| **Model 1 - Early childhood** |  |  |  |  |  |  |  |  |
| Internal assets | .16 (.09, .23) | < .01* | .10 (.02, .17) | .01* | -.09 (-.15, -.02) | .01* | -.10 (-.16, -.03) | < .01* |
| External assets | .04 (-.02, .11) | .16 | .07 (.01, .13) | .02* | .02 (-.04, .08) | .56 | -.04 (-.09, .02) | .25 |
| **Model 2 - Late childhood** |  |  |  |  |  |  |  |  |
| Internal assets | .15 (.08, .23) | < .01* | .09 (.01, .17) | .03* | -.07 (-.14, .00) | .06 | -.12 (-.19, -.05) | < .01* |
| External assets | .13 (.06, .19) | < .01* | .16 (.09, .22) | < .01* | -.08 (-.15, -.02) | .01* | -.16 (-.22, -.09) | < .01* |
| **Model 3 - Adolescence** |  |  |  |  |  |  |  |  |
| Internal assets | .18 (.11, .23) | < .01* | .14 (.08, .20) | < .01* | -.10 (-.16, -.03) | < .01* | -.18 (-.24, -.12) | < .01* |
| External assets | .13 (.06, 19) | < .01* | .15 (.09, .22) | < .01* | -.05 (-.11, .01) | .12 | -.09 (-.15, -.03) | < .01* |
| *Notes*. Estimates are z-standardized. Internal and External dimension scales were entered together in all models. Models were adjusted for covariates: sex, socioeconomic status, parent origin, family type, externalizing and internalizing problems in early childhood.  ^a^Data were compiled from the final master file of the Quebec Longitudinal Study of Child Development (1998-2020), ©Gouvernement du Québec, Institut de la statistique du Québec.  * *p* < .05 after false-discovery rate correction. | | | | | | | | |

| Table S6. Multiple Regression Models Predicting Mental Health Outcomes from Asset Categories by Developmental Periods | | | | | | | | |
| --- | --- | --- | --- | --- | --- | --- | --- | --- |
|  | **Life satisfaction**  **19-21 years** | | **Happiness**  **19-21 years** | | **Anxiety symptoms**  **20-22 years** | | **Depressive symptoms**  **20-22 years** | |
|  | **B (95% CI)** | **p-value** | **B (95% CI)** | **p-value** | **B (95% CI)** | **p-value** | **B (95% CI)** | **p-value** |
| **Model 1 - Early childhood** |  |  |  |  |  |  |  |  |
| *Internal assets* |  |  |  |  |  |  |  |  |
| Commitment to Learning | .03 (-.04, .09) | .47 | .01 (-.06, .08) | .73 | .04 (-.03, .11) | .24 | -.01 (-.07, .06) | .83 |
| Responsibility | .05 (-.03, .14) | .21 | .05 (-.04, .13) | .28 | -.03 (-.10, .04) | .41 | -.02 (-.10, .06) | .56 |
| Social Competencies | .13 (.05, .21) | <.01* | .08 (-.01, .16) | .08 | -.08 (-.16, -.01) | .03 | -.09 (-.17, -.01) | .02 |
| *External assets* |  |  |  |  |  |  |  |  |
| Support | .06 (-.01, .13) | .09 | .07 (-.01, .14) | .05 | -.00 (-.07, .07) | .94 | -.04 (-.11, .02) | .21 |
| Boundaries and Expectations | -.03 (-.09, .03) | .31 | -.01 (-.07, .05) | .77 | -.00 (-.06, .06) | .96 | .00 (-.06, .06) | .99 |
| Constructive Use of Time | .01 (-.05, .07) | .78 | .01 (-.05, .07) | .79 | .01 (-.05, .08) | .66 | -.00 (-.06, .06) | .90 |
| **Model 2 - Late childhood** |  |  |  |  |  |  |  |  |
| *Internal assets* |  |  |  |  |  |  |  |  |
| Commitment to Learning | .07 (-.01, .16) | .08 | .05 (-.04, .14) | .26 | -.04 (-.11, .04) | .31 | -.06 (-.14, .02) | .12 |
| Responsibility | -.00 (-.09, .08) | .92 | .01 (-.08, .10) | .82 | .02 (-.07, .10) | .68 | .02 (-.06, .10) | .64 |
| Social Competencies | .13 (.01, .24) | .04 | .07 (-.05, .19) | .27 | -.07 (-.17, .03) | .16 | -.11 (-.22, -.01) | .04 |
| *External assets* |  |  |  |  |  |  |  |  |
| Support | .09 (.03, .16) | .01* | .11 (.04, .19) | < .01* | -.07 (-.14, -.00) | .04 | -.09 (-.15, -.02) | .01* |
| Boundaries and Expectations | -.04 (-.10, .03) | .29 | -.03 (-.09, .04) | .37 | .07 (.01, .13) | .03 | .02 (-.04, .09) | .51 |
| Constructive Use of Time | .07 (.00, .13) | .04 | .08 (.02, .15) | .01* | -.06 (-.13, -.00) | .05 | -.11 (-.17, -.05) | < .01* |
| **Model 3 - Adolescence** |  |  |  |  |  |  |  |  |
| *Internal assets* |  |  |  |  |  |  |  |  |
| Commitment to Learning | .05 (-.03, .12) | .21 | .01 (-.06, .08) | .71 | .06 (-.01, .14) | .08 | .03 (-.04, .10) | .42 |
| Responsibility | -.05 (-.11, .01) | .13 | -.04 (-.10, .02) | .17 | .05 (-.00, .11) | .07 | .05 (-.01, .11) | .12 |
| Social Competencies | .14 (.08, .21) | < .01* | .14 (.07, .20) | <.01* | -.19 (-.26, -.12) | < .01* | -.23 (-.30, -.16) | < .01* |
| *External assets* |  |  |  |  |  |  |  |  |
| Support | .21 (.12, .31) | < .01* | .23 (.14, .33) | < .01* | -.08 (-.16, .00) | .06 | -.13 (-.21, -.05) | < .01* |
| Boundaries and Expectations | -.11 (-.19, -.03) | .01* | -.11 (-.18, -.03) | .01* | .07 (-.01, .14) | .09 | .06 (-.00, .13) | .07 |
| Constructive Use of Time | -.00 (-.09, .08) | .92 | .00 (-.07, .08) | .90 | -.01 (-.07, .06) | .84 | -.02 (-.09, .05) | .64 |
| *Notes*. Estimates are z-standardized. Positive Identity and Safety were not included because they were unavailable at more than one time point, therefore we could not combine assessments into developmental periods. All asset categories were entered together in each model. Models were adjusted for covariates (sex, socioeconomic status, parental origin, family type, externalizing and internalizing problems in early childhood).  Data were compiled from the final master file of the Quebec Longitudinal Study of Child Development (1998-2020), ©Gouvernement du Québec, Institut de la statistique du Québec.  * *p* < .05 after false-discovery rate correction. | | | | | | | | |

| Table S7. SLCMA-Selected Life-Course Hypothesis for Internal and External Dimension Scales by Developmental Periods | | | | | | | | | | | | |
| --- | --- | --- | --- | --- | --- | --- | --- | --- | --- | --- | --- | --- |
|  | Life satisfaction 19-21 years | | | Happiness 19-21 years | | | Anxiety symptoms 20-22 years | | | Depression symptoms 20-22 years | | |
|  | Step 1 | | Step 2 | Step 1 | | Step 2 | Step 1 | | Step 2 | Step 1 | | Step 2 |
|  | Model | R^2^ | Estimate  (95% CI) | Model | R^2^ | Estimate  (95% CI) | Model | R^2^ | Estimate  (95% CI) | Model | R^2^ | Estimate  (95% CI) |
| Internal assets | Recency | .053 | **.034**  **(.027, .042)** | Recency | .009 | **.028**  **(.012, .036)** | Recency | .012 | **-.017**  **(-.025, -.009)** | Recency | .023 | **-.031**  **(-.039, -.023)** |
| External assets | Recency | .033 | **.029**  **(.021, .037)** | Recency | .040 | **.032**  **(.023, .040)** | Late childhood | .009 | **-.097**  **(-.145, -.040)** | Late childhood | .022 | **-.176**  **(-.224, -.125)** |
| *Notes.* Step 1 corresponds to the Least Angle Regression (LARS) models. Step 2 corresponds to the post-selection inference. Estimates in bold were significant according to a Benjamini-Hochberg FDR-adjusted *p-*value < .05 considering the six models. R^2^ represents the proportion of variance in mental health outcomes explained by the developmental assets, according to the selected hypothesis and after adjusting for covariates (sex, socioeconomic status, parental origin, family type, externalizing and internalizing problems in early childhood). For the recency hypothesis, the estimate from step 2 represents the change in the mental health outcome z-score for a one-point increase in exposure, weighted by the temporal proximity of exposure to the outcome. For the time-specific, sensitive period hypothesis, the estimate reflects the difference in the outcome z-score between individuals exposed vs. unexposed at this specific age.  Abbreviations: SLCMA = Structured Life-course Modeling Approach; CI = confidence intervals.  Data were compiled from the final master file of the Quebec Longitudinal Study of Child Development (1998-2020), ©Gouvernement du Québec, Institut de la statistique du Québec. | | | | | | | | | | | | |

| Table S8. SLCMA-Selected Life-Course Hypothesis for Each Internal and External Asset Category by Developmental Periods | | | | | | | | | | | | |
| --- | --- | --- | --- | --- | --- | --- | --- | --- | --- | --- | --- | --- |
|  | Life satisfaction 19-21 years | | | Happiness 19-21 years | | | Anxiety symptoms 20-22 years | | | Depression symptoms 20-22 years | | |
|  | Step 1 | | Step 2 | Step 1 | | Step 2 | Step 1 | | Step 2 | Step 1 | | Step 2 |
|  | Model | R^2^ | Estimate  (95% CI) | Model | R^2^ | Estimate  (95% CI) | Model | R^2^ | Estimate  (95% CI) | Model | R^2^ | Estimate  (95% CI) |
| Internal assets | | | | | | | | | | | | |
| Commitment to Learning | **~~-~~** | **~~-~~** | **~~-~~** | **~~-~~** | **~~-~~** | **~~-~~** | ~~-~~ | ~~-~~ | ~~-~~ | ~~-~~ | ~~-~~ | ~~-~~ |
| Responsibility | **~~-~~** | **~~-~~** | **~~-~~** | - | - | - | ~~-~~ | ~~-~~ | ~~-~~ | ~~-~~ | ~~-~~ | ~~-~~ |
| Social Competencies | Recency | .057 | **.032**  **(.025, .038)** | - | - | - | Adolescence | .010 | **-.098**  **(-.129, -.046)** | Adolescence | .001 | **-.082**  **(-.144, -.019)** |
|  |  |  |  |  |  |  |  |  |  | Recency | .064 | **-.016**  **(-.030, -.002)** |
| External assets | | | | | | | | | | | | |
| Support | Adolescence | .008 | **.124**  **(.043, .154)** | Adolescence | .016 | **.130**  **(.086, .162)** | ~~-~~ | ~~-~~ | ~~-~~ | Recency | .027 | **-.023**  **(-.030, -.016)** |
| Boundaries and Expectations | ~~-~~ | ~~-~~ | ~~-~~ | Recency | .001 | .009  (-.024, .016) | ~~-~~ | ~~-~~ | ~~-~~ | ~~-~~ | ~~-~~ | ~~-~~ |
| Constructive Use of Time | ~~-~~ | ~~-~~ | ~~-~~ | Recency | .008 | **.017**  **(.006, .024)** | ~~-~~ | ~~-~~ | ~~-~~ | Late childhood | .012 | **-.095**  **(-.130, -.052)** |
| *Notes.* Dashes indicate SLCMA models that were not tested because multiple regression results showed no significant association between the asset category and the outcome. Step 1 corresponds to the Least Angle Regression (LARS) models. Step 2 corresponds to the post-selection inference. Estimates in bold were significant according to a Benjamini-Hochberg FDR-adjusted *p-*value < .05 considering the nine models. R^2^ represents the proportion of variance in mental health outcomes explained by the developmental assets, according to the selected hypothesis and after adjusting for covariates (sex, socioeconomic status, parental origin, family type, externalizing and internalizing problems in early childhood). For the recency hypothesis, the estimate from step 2 represents the change in the mental health outcome z-score for a one-point increase in exposure, weighted by the temporal proximity of exposure to the outcome. For the time-specific, sensitive period hypothesis, the estimate reflects the difference in the outcome z-score between individuals exposed vs. unexposed at this specific age.  Abbreviations: SLCMA = Structured Life-course Modeling Approach; CI = confidence intervals.  Data were compiled from the final master file of the Quebec Longitudinal Study of Child Development (1998-2020), ©Gouvernement du Québec, Institut de la statistique du Québec. | | | | | | | | | | | | |

# Figure S1. Multiple Regression Models Predicting Mental Health Outcomes from the Internal and External Dimension Scales in the QLSCD^a^ by Developmental Periods

*Notes*. Internal and External dimension scales were entered together in the same model; their estimates are thus independent. Models were also adjusted for covariates (sex, socioeconomic status, parental origin, family type, externalizing and internalizing problems in early childhood). Estimates are z-standardized.

Abbreviations: QLSCD = Quebec Longitudinal Study of Child Development.

^a^Data were compiled from the final master file of the Quebec Longitudinal Study of Child Development (1998–2018), ©Gouvernement du Québec, Institut de la statistique du Québec.

* *p* < .05 after false-discovery rate correction.

# Figure S2. Multiple Regression Models Predicting Mental Health Outcomes from each Asset Categories in the QLSCD^a^ by Developmental Periods

*Notes*. Positive Identity and Safety were not included as they were unavailable at more than one time point, which precluded combining assessments into developmental periods. All eight asset categories were entered together in the same model; their estimates are thus independent. Models were also adjusted for covariates (sex, socioeconomic status, parental origin, family type, externalizing and internalizing problems in early childhood). Estimates are z-standardized.

Abbreviations: QLSCD = Quebec Longitudinal Study of Child Development.

^a^Data were compiled from the final master file of the Quebec Longitudinal Study of Child Development (1998–2018), ©Gouvernement du Québec, Institut de la statistique du Québec.

* *p* < .05 after false-discovery rate correction.

# References

Bentler, Peter M. (1990). Comparative fit indexes in structural models. *Psychological Bulletin*, *107*(2), 238‑246.

Briggs, S. R., & Cheek, J. M. (1986). The role of factor analysis in the development and evaluation of personality scales. *Journal of Personality*, *54*(1), 106‑148. https://doi.org/10.1111/j.1467-6494.1986.tb00391.x

Hu, L., & Bentler, P. M. (1999). Cutoff criteria for fit indexes in covariance structure analysis : Conventional criteria versus new alternatives. *Structural Equation Modeling: A Multidisciplinary Journal*, *6*(1), 1‑55. https://doi.org/10.1080/10705519909540118

MacCallum, Robert C., Browne, Michael W., & Sugawara, Hazuki M. (1996). Power analysis and determination of sample size for covariance structure modeling. *Psychological Methods*, *1*(2), 130‑149.

Thibault, M., Matte-Landry, A., Bilodeau-Houle, A., Boivin, M., Geoffroy, M.-C., Côté, S. M., & Ouellet-Morin, I. (in press). Predicting early adulthood outcomes from adolescent developmental assets: operationalization and longitudinal analysis of internal and external resources. *Applied Developmental Science*. https://doi.org/10.1080/10888691.2026.2675290

Tucker, L. R., & Lewis, C. (1973). A reliability coefficient for maximum likelihood factor analysis. *Psychometrika*, *38*(1), 1‑10. https://doi.org/10.1007/BF02291170
